# Comparative Efficacy and Safety of Chinese Patent Medicines for Cervical High-risk Human Papillomavirus Infection: A Bayesian Network Meta-analysis

**DOI:** 10.7150/jca.86043

**Published:** 2023-07-31

**Authors:** Chengli Bin, Hanzhi Zhong, Jing Mo, Zhenyi Wang, Maoya Li, Shaobin Wei

**Affiliations:** 1 Hospital of Chengdu University of Traditional Chinese Medicine, Chengdu, China.; 2 Graduate School, Chengdu University of Traditional Chinese Medicine, Chengdu, China.

**Keywords:** Chinese patent medicine, high-risk human papillomavirus infection, network Meta-analysis, randomized controlled trial, clinical efficacy

## Abstract

**Background:** Many Chinese patent medicines have been reported to show anti-tumor cell effects on cervical cancer. To estimate the comparative effectiveness and safety of Chinese patent medicines for cervical high-risk human papillomavirus (HR-HPV) infection.

**Methods:** Randomized controlled trials (RCTs) of Chinese patent medicines for HR-HPV infection were searched in eight databases until 31 August 2022, and two review authors independently extracted data and assessed the risk of bias. Outcomes concerning efficacy were evaluated as odds ratio (OR) and 95% credible intervals (CrI) utilizing R 4.1.2 and Stata 16.1. The protocol was registered on the International Prospective Register of Systematic Reviews (CRD42022351120).

**Results:** We ultimately identified 60 RCTs that involved 5,951 participants and 8 interventions. Chinese patent medicines combined with recombinant human interferon (rhIFN) have better treatment effects than rhIFN alone. The results showed that Baofukang suppository (BFK) combined with rhIFN is greater for the rate of HR-HPV clearance follow-up at 6 months (SUCRA = 78.16%). Kushen gel (KS) combined with rhIFN ranked first for the rate of HR-HPV clearance after treatment (SUCRA = 90.77%). Furthermore, KS + rhIFN is most likely to be the best intervention for improving the clinical effectiveness rate (SUCRA = 87.39%). Adverse reactions were not statistically significant in BFK + rhIFN versus BFK, BFK + rhIFN versus rhIFN, and BFK versus rhIFN.

**Conclusion:** The combination of Chinese patent medicines with rhIFN may demonstrate a higher efficacy than rhIFN alone in clearing the virus and improving cervical symptoms. Weighing with the clinical comprehensive efficacy, BFK + rhIFN and KS + rhIFN maybe the optimal treatments for cervical HR-HPV infection. However, more high-quality randomized controlled trials are needed in the future to further confirm the efficacy and safety of proprietary Chinese medicines for cervical HR-HPV infection.

## Introduction

Cervical cancer is the second largest malignant tumor in female malignancy after breast cancer 1, and the mortality rate ranks first 2. There were an estimated 604,000 new cases of cervical cancer and 342,000 deaths globally in 2020, seriously affecting women's health 3. It is worth that the incidence of cervical cancer in developing countries has been on the rise in recent years due to the lack of effective prevention methods 4. Studies have shown that the incidence and death of cervical cancer have been on the rise in the past 10 years and the next 10 years, and are one of the public problems facing the world 5. Most women with high-risk human papillomavirus (HR-HPV) infection can be cleared from the body through autoimmune ability, but 5%~10% of infected women are still unable to clear HR-HPV, resulting in persistent HR-HPV infection. The natural evolution process from HR-HPV lasting ≥ 2 years to precancerous lesions and finally developing into cervical cancer takes about 5~15 years 6. In the long natural evolution of the disease, blocking the persistent infection of HR-HPV is currently the first measure to prevent cervical cancer.

In 2021, the International Federation of Gynecology and Obstetrics (FIGO) proposed tertiary prevention measures for cervical cancer, but mainly for uninfected people and people who have already developed cervical intraepithelial neoplasia (CIN) 2 and CIN 3 7. At present, western medicine clinical treatment methods for HPV infection include drug treatment, physical therapy, surgery and vaccines, and topical interferon suppositories are mostly used for cervical HR-HPV persistent infection 8. Recombinant human interferon is a commonly used antiviral drug, which can effectively inhibit the replication of viral genetic material and enhance the patient's immune system, which can achieve better and shorter efficacy. However, the recurrence rate is high after discontinuation, and long-term medication may also affect patient tolerance and compliance 9. Detoxification therapy in Chinese medicine has been conducted recently 10. Many molecules extracted from herbs and natural products have been found in the laboratory to be potential to prevent cancer 11. Some studies 12-15 have reported that Chinese medicinal preparations can achieve anti-tumor, anti-virus and insecticidal effects on HPV-infected patients, and play an important role in regulating local immunity.

At present, local medicines with good effects at home and abroad still cannot meet the clinical needs, Chinese patent medicine combined with interferon seems to be expected to improve the long-term efficacy of HR-HPV infection. There are literatures report on systematic reviews of proprietary Chinese medicines alone or in combination with recombinant human interferon for the treatment of HR-HPV infection, such as Baofukang suppository (BFK) 16, 17 and Kushen gel (KS) 18. For the treatment of HR-HPV infection, there are many types of proprietary Chinese medicines, but there is still no comparison of their efficacy. Therefore, it is difficult to evaluate the efficacy and safety of several Chinese patent medicines alone and in combination with recombinant human interferon in treating HR-HPV. To gain insight into the strengths and weaknesses of various interventions, we are going to rank them according to the direct or indirect evidence available through Bayesian network meta-analysis (NMA). This study aims to provide sufficient clinical evidence for the most appropriate evidence-based medical approach in traditional Chinese medicine and can provide a certain reference value for the formulation of clinical guidelines and clinical practice.

## Methods

### Protocol and Registration

The procedure of current NMA was conducted in accordance with the Preferred Reporting Items for Systematic reviews and Meta-Analyses (PRISMA) guidelines “NMA extended version” 19. The completed PRISMA check list was presented as **Supplementary S1**. The protocol was registered on the International Prospective Registration of Systematic Reviews (PROSPERO) (Number CRD42022351120). There was no ethical approval or consent required for any of the analyses because they were based on previously published studies.

### Search Strategies

The randomized controlled trials (RCTs) of Chinese patent medicine alone or in combination with recombinant human interferon for HR-HPV infection included were recruited through PubMed, Embase, Cochrane Central Register of Controlled Trials, Web of Science, Chinese Biomedical Databases (CBM), China National Knowledge Infrastructure (CNKI), Chinese Scientific Journals Database (VIP), and Wanfang data from the start of database construction until August 31, 2022. Medical Subject Heading (MeSH) and keyword search terms included were “Chinese herbal”, “Traditional Chinese Medicine”, “Chinese patent medicines”, “high-risk human papillomavirus infection”, “high-risk HPV infection”, “cervical intraepithelial neoplasia”. Besides, the searched Chinese patent medicines were all listed in the Catalogue of Drugs for Basic National Medical Insurance (The 2018 Edition). The English database search strategy was provided in **Supplementary S2**. Further eligible studies were identified by manually searching the reference lists of relevant meta-analyses and review articles.

### Inclusion Criteria

We included published RCTs based on the predetermined selection rules that met the following PICOS criterion:

1) Population: Women diagnosed with oncogenic HR-HPV positive but cytology negative or cytology-atypical squamous cells of undetermined significance (ASC-US), or low-grade squamous intraepithelial lesions (LISL), or CIN 1. The definition, screening of HR-HPV and cervical cytology was according to American Society for Colposcopy and Cervical Pathology (ASCCP) 20, the American College of Obstetricians and Gynecologists (ACOG) guidelines 21 and Chinese Society for Colposcopy and Cervical Pathology of China Healthy Birth Science Association (CSCCP) 22. Without age, race or region restriction.

1. Intervention: The experiment group involve Chinese patent medicines combined with recombinant human interferon, Chinese patent medicines only. Both Chinese patent medicines and recombinant human interferon are transvaginal medications.

3) Comparison: The control group consist of recombinant human interferon alone or Chinese patent medicines.

4) Outcomes: The primary outcome was the rate of HR-HPV clearance follow-up at 6 months (total sample size of patients turned negative follow-up at 6 months / total sample size of patients * 100%). The secondary outcomes were the rate of HR-HPV clearance after treatment and clinical effectiveness rate.

5) Study design: We confined our study design to RCTs published with Chinese or English restriction.

### Exclusion Criteria

Studies not adhering to the inclusion criteria were excluded. Other exclusion criteria were:

1) Nonrandomized controlled studies such as case-control studies, cohort studies.

2) Trials not including the relevant outcomes.

3) Trials that did not clearly identify HR-HPV.

4) Participants with high-grade CIN 2, CIN 3.

5) Data not available or cannot be extracted.

6) Interventions include surgery or physiotherapy.

7) The duration of treatment is more than 6 months or less than half a month.

8) Sample size of the experiment group or control group is less than 10.

### Literature Screening and Data Extraction

Two investigators (BCL and MJ) screened the titles, abstracts and full text of retrieved citations independently to identify potentially eligible trials. Any disagreements were discussed between researchers until a consensus was reached. We implemented data a extraction form the collection relevant data for included trial. The following information was recorded from the included RCTs: basic publication information (first author name, publication year), characteristics of the participants (sample size, baseline age, duration of disease), intervention, intervention duration and outcomes.

### Quality Assessment

Two investigators (BCL and MJ) independently conducted quality assessment. Any disagreement was resolved through discussion or judged by an expert as a third author (WSB). We assessed risk of bias of individual studies using the Cochrane Risk of Bias Tool 23. The quality evaluation of the included RCTs focused on the following domains: selection bias (sequence generation and allocation concealment), performance bias (blinding of patients and personnel), detection bias (blinding of outcome assessors), attrition bias (incomplete outcome data), reporting bias (selective reporting), and other biases. Each trial received a study level score of low, high, or unclear risk of bias for each domain.

We used GRADE (grading of recommendations assessment, development, and evaluation) to evaluate the quality of the evidence for each outcome measure. For direct evidence, we evaluate the quality from five aspects (risk of bias, inconsistency, inaccuracy, indirectness, imprecision and publication bias) 24. For the network meta-analysis, we added accuracy and inconsistency to evaluate 25.

### Statistical Analyses

The key assumptions of a network meta-analysis are transitivity, homogeneity and consistency. To determine whether a network meta-analysis was appropriate, the clinical setting and characteristics of trials (considering age, duration of disease, intervention duration, duration of follow-up, and year of publication) reporting each drug class were evaluated. To determine the degree of fit of the model, the deviance information criterion (DIC) between the fixed-effect and random-effect models was used 26. Both models were consistent enough for adoption if the difference in the DIC of <5. However, given the clinical heterogeneity between the studies contributing to an outcome, the results of random effects models were presented. A model with a smaller DIC was adopted if the difference was >5. Heterogeneity was quantitatively determined using I^2^, with I^2^ < 50% indicating no statistical heterogeneity 27. When a loop connecting three arms existed, we applied the split-node method by reporting its Bayesian p-value to assess inconsistency 28. If the p-value was > 0.05, the direct and indirect evidence was considered to be consistent. The odds ratio (OR) was used to calculate the treatment effect with 95% credible intervals (CrI) for dichotomous data (e.g, HPV cleared or non-cleared) 29. All interventions were integrated network meta-analysis within a Bayesian framework based on 200,000 iterations to produce the outputs and 5,000 burn-in iterations to allow convergence and annealing algorithm.

In order to demonstrate direct and indirect comparative relationships among different interventions, we developed evidence networks 30. The surface under the cumulative ranking curve (SUCRA) values was used to visualize the pros and cons of interventions in each outcome measure 31. We also produced comparison adjusted funnel plots to evaluate the presence of small study effects or publication biases for each outcome 32. We made a subgroup analysis for the outcome of the rate of HR-HPV clearance according to two follow-up time points: after treatment, and follow-up at 6 months. Additionally, the cluster analysis was conducted for choosing the optimal treatments under two independent outcomes simultaneously 33. All Bayesian NMA was conducted using the R 4.2.1 software and the gemtc package. The Stata 16.1 software was used for evidence networks, funnel plots and cluster graphs. The RevMan 5.4 software was used for direct pairwise meta-analysis.

## Results

### Study selection and characteristics

**Figure 1:**
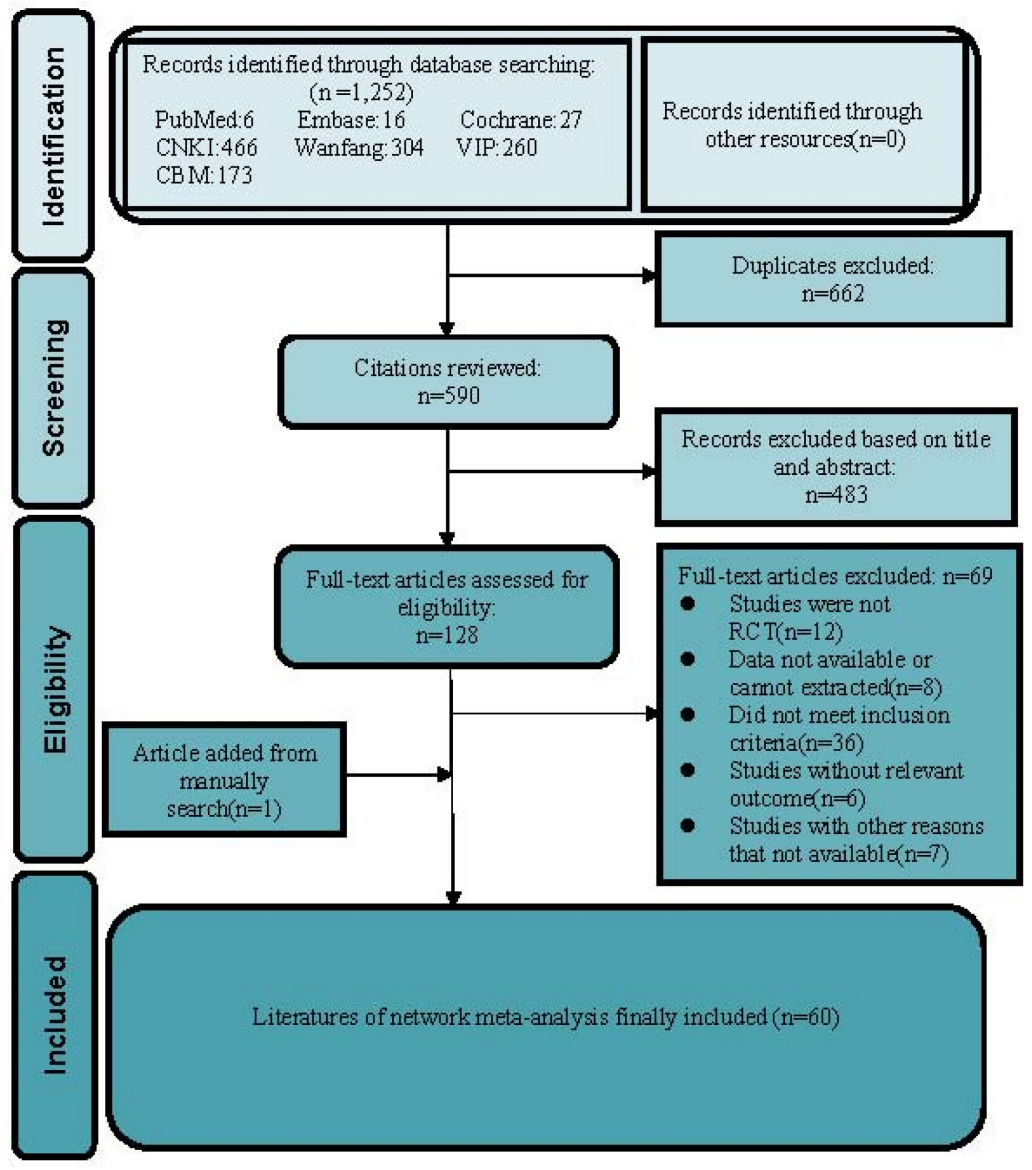
Literature review flowchart. CNKI = China National Knowledge Infrastructure database; VIP = Chinese scientific Journal database; CBM = Chinese Biomedical Literature database; RCT = Randomized controlled trial.

We identified 1,252 records from the initial title and abstract screening and retrieved and reviewed 107 reports in full text. We retrieved relevant articles by hand-search to ensure we didn't miss eligible research by omitting the electronic database. We found one RCT 34 that met the inclusion criteria by reading several meta-analyses 16-18, 35 of proprietary Chinese medicines for HPV infection. Eventually, 60 RCTs involving 5,951 participants met the inclusion criteria and enrolled to receive 8 different treatments including recombinant human interferon (rhIFN), recombinant human interferon combined with Baofukang suppository (BFK + rhIFN), Compound seabuckthorn seed oil suppository (SJZ + rhIFN), Kushen gel (KS + rhIFN), Zhimikang suppository (ZMK + rhIFN), Baofukang suppository (BFK), Compound seabuckthorn seed oil suppository (SJZ), Kushen gel (KS). The literature screening process was illustrated in **Figure 1**. All of the 60 RCTs were published as Chinese language articles from 2011 to 2022. All the eligible Chinese patent medicines and rhIFN were taken vaginally, and the duration of intervention was from 1 to 3 months. A reference list of the included RCTs was shown in **Supplementary S3**. We considered a network meta-analysis to be appropriate due to the comparability of patients involved, outcome measures, inclusion criteria, and exclusion criteria. The detailed characteristics of 60 included researches were demonstrated in **Supplementary S4**. More details of the Chinese patent medicines product (raw materials, labeled efficacy, indications, extraction procedure) were presented in **Supplementary S5,** which is convenient for gynecologists and pharmacologists to acquire comprehensive information of the included Chinese patent medicines.

### Quality Assessment

Most trials (51.67%) described the stochastic methods in detail as random numbers or random lottery. None of those trials mentioned blinding of participants and personnel. All trials reflected a low risk of bias in incomplete outcome data and other biases with sufficient original data. The selection and reporting bias of all studies were unclear risk. Briefly, the risk of bias was moderate in most of the evaluation entries. Full details regarding risk of bias for each RCT were shown in **Supplementary S6**. All of the evidence quality was low or very low. **Supplementary S7**-**S9** presented the GRADE assessment for three outcomes.

### Pairwise meta-analysis

**Figure 2:**
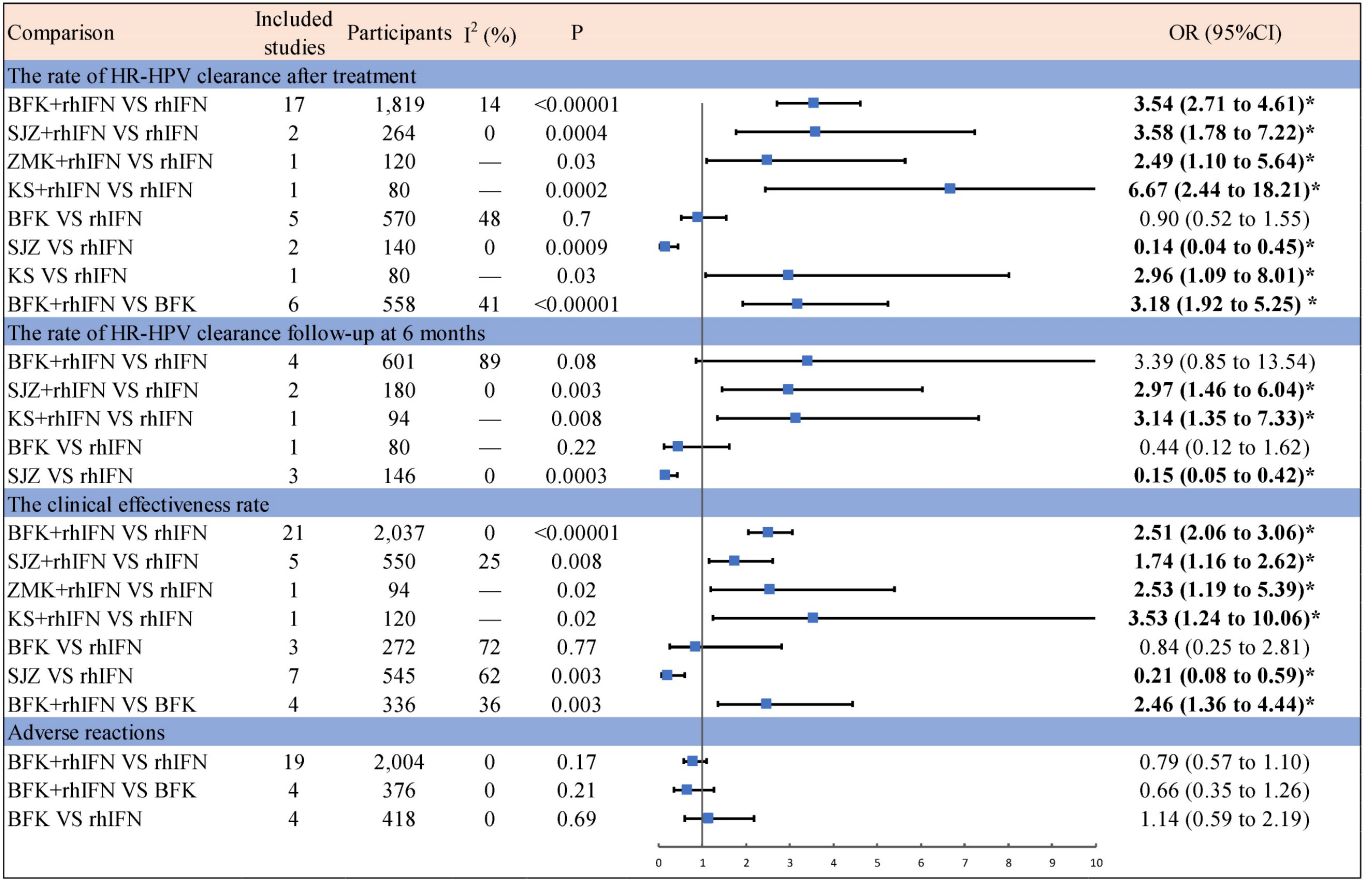
Results of pairwise Meta-Analysis and heterogeneity estimates. OR = odds ratio; CI = confidence interval; Bold and marked with * indicates statistical significance (*p* < 0.05); BFK = Baofukang suppository; SJZ = Compound seabuckthorn seed oil suppository; KS = Kushen gel; ZMK = Zhimikang suppository; rhIFN = recombinant human interferon.

**Figure 2** presented the results of the pairwise meta-analysis and heterogeneity estimates. The results of the pairwise meta-analysis showed that four Chinese patent medicines (BFK, SZJ, ZMK, KS) compared with rhIFN were better than only rhIFN for the HR-HPV clearance rate and the clinical effectiveness rate. BFK + rhIFN was better than only BFK for the HR-HPV clearance rate after treatment and the clinical effectiveness rate. SZJ was worse than rhIFN for the HR-HPV clearance rate and the clinical effectiveness rate. Adverse reactions were not statistically significant in BFK + rhIFN versus BFK, BFK + rhIFN versus rhIFN, and BFK versus rhIFN.

### Network meta-analysis

**Figure 3:**
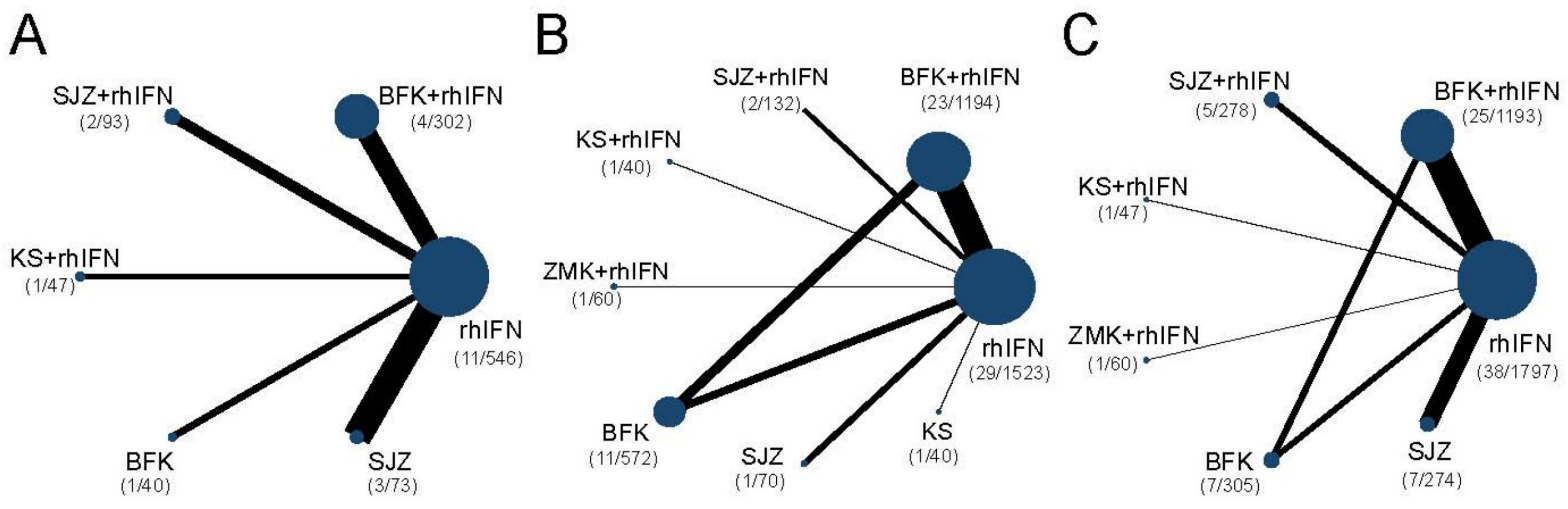
Network diagrams of comparisons on different outcomes of treatments. SUCRA = Surface Under the Cumulative Ranking curve; BFK = Baofukang suppository; SJZ = Compound seabuckthorn seed oil suppository; KS = Kushen gel; ZMK = Zhimikang suppository; rhIFN = recombinant human interferon. **(A)** The rate of HR-HPV clearance follow-up at 6 months; **(B)** The rate of HR-HPV clearance after treatment; **(C)** The clinical effectiveness rate.

The networks were presented in **Figure 3**. Briefly, all outcomes were recombinant human interferon-centric, with the rate of HR-HPV clearance follow-up at 6 months with no closed loop, and for the other two outcomes with one closed loop. **Supplementary S10** presented the DIC values and I-square of the fixed-effect model and random-effect model. The DICs of the rate of HR-HPV clearance after treatment and clinical effectiveness rate were similar, and the rate of HR-HPV clearance follow-up at 6 months was smaller in the random-effect model. Therefore, the random-effect model of the three outcomes was used for data analysis in this study. There was no statistical heterogeneity for three outcomes with I^2^ < 50%.

### Primary outcome

#### The rate of HR-HPV clearance follow-up at 6 months

There were 6 interventions, of which rhIFN (11 RCTs, 526 participants) was the most studied, followed by BFK + rhIFN (4 RCTs, 302 participants). The network plot for the primary outcome of the rate of HR-HPV clearance follow-up at 6 months appeared in **Figure 3**. The results of the network meta-analysis indicated that BFK + rhIFN (OR: 36.54, 95%CrI: 3.99 to 570.11), SJZ + rhIFN (OR: 34.93, 95%CrI: 2.46 to 753.49), KS + rhIFN (OR: 34.50, 95%CrI: 1.33 to 1319.18), rhIFN (OR: 10.72, 95%CrI: 1.77 to 95.80) were better than SJZ. There was no statistically significant difference between the other outcomes (see in **Table 1**). According to the ranking probabilities of **Table 1** and **Supplementary S11**, BFK + rhIFN (SUCRA = 78.16%) was the first-rank combination in improving the HR-HPV clearance rate follow-up at 6 months, followed by SJZ + rhIFN (SUCRA = 75.40%) and KS + rhIFN (SUCRA = 73.17%).

### Secondary Outcomes

#### The rate of HR-HPV clearance after treatment

There were 24 RCTs reported the rate of HR-HPV clearance after treatment including 8 interventions (see in **Figure 3**). According to SUCRA probability results (**Table 2, Supplementary S11**), KS + rhIFN was the most likely to become the best option for improving the HR-HPV clearance rate after treatment (SUCRA = 90.77%), followed by BFK + rhIFN (SUCRA = 72.15%) and SJZ + rhIFN (SUCRA = 70.04%).

#### The clinical effectiveness rate

A total of 11 RCTs reported the clinical effectiveness rate (see in **Figure 3**), which was higher in KS + rhIFN (OR: 3.66, 95%CrI: 1.18 to 12.15), BFK + rhIFN (OR: 2.56, 95%CrI: 2.05 to 3.25), ZMK + rhIFN (OR: 2.58, 95%CrI: 1.03 to 6.80), SJZ + rhIFN (OR: 1.77, 95%CrI: 1.15 to 2.71) than in those using only rhIFN (see in **Table 3**). On the basis of SUCRA probability results (**Table 3, Supplementary S11**), KS + rhIFN (SUCRA = 87.39%) was the optimum therapeutic measure in improving the clinical effectiveness rate, BFK + rhIFN (SUCRA = 78.61%) was number two, and ZMK + rhIFN (SUCRA = 75.78%) was the third.

### Cluster analysis

**Figure 4:**
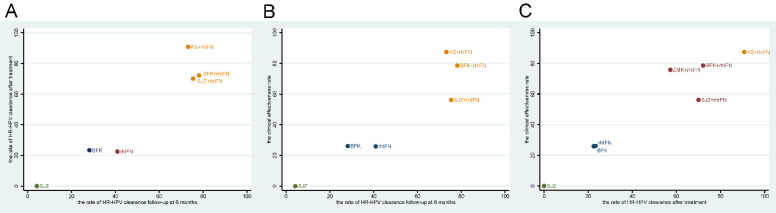
Cluster diagram. Measures of the same color in the figure have similar efficacy, and the closer they are to the upper right, the higher their overall ranking. BFK = Baofukang suppository; SJZ = Compound seabuckthorn seed oil suppository; KS = Kushen gel; ZMK = Zhimikang suppository; rhIFN = recombinant human interferon. **(A)** The rate of HR-HPV clearance follow-up at 6 months and the rate of HR-HPV clearance after treatment; **(B)** The rate of HR-HPV clearance follow-up at 6 months and the clinical effectiveness rate; **(C)** The rate of HR-HPV clearance after treatment and the clinical effectiveness rate.

Based on the SUCRA values of the original network analysis results, we used cluster analysis to obtain the optimum treatments under the rate of HR-HPV clearance follow-up at 6 months, the rate of HR-HPV clearance follow-up after treatment and the clinical effectiveness rate. There were seven interventions participated in the analysis, the results showed that the comprehensive clinical efficacy of proprietary Chinese medicine combined with recombinant human interferon was better than that of interferon or proprietary Chinese medicine alone. Besides, BFK + rhIFN and KS + rhIFN were the best options, while SJZ + rhIFN had a poor effect on improving clinical symptoms. The cluster diagrams were presented in **Figure 4**.

### Adverse reactions

Twenty-seven RCTs mentioned adverse effects after medication, of which 20 were BFK + rhIFN versus rhIFN, 4 were BFK + rhIFN versus BFK, and 3 were BFK compared with rhIFN alone. And no network meta-analysis was performed for adverse reactions. The most common adverse reactions reported in the included trials were local vaginal symptoms, namely itching, dryness, bleeding, swelling, sting, and increased vaginal discharge; gastrointestinal reactions involving nausea, vomiting, and abdominal distension; allergic reactions including rash and itching of the skin; other symptoms involving fever, menstrual disorders, and liver dysfunction. The details of adverse reactions were presented in **Supplementary S12**.

### Publication Bias and Consistency Tests

**Figure 5:**
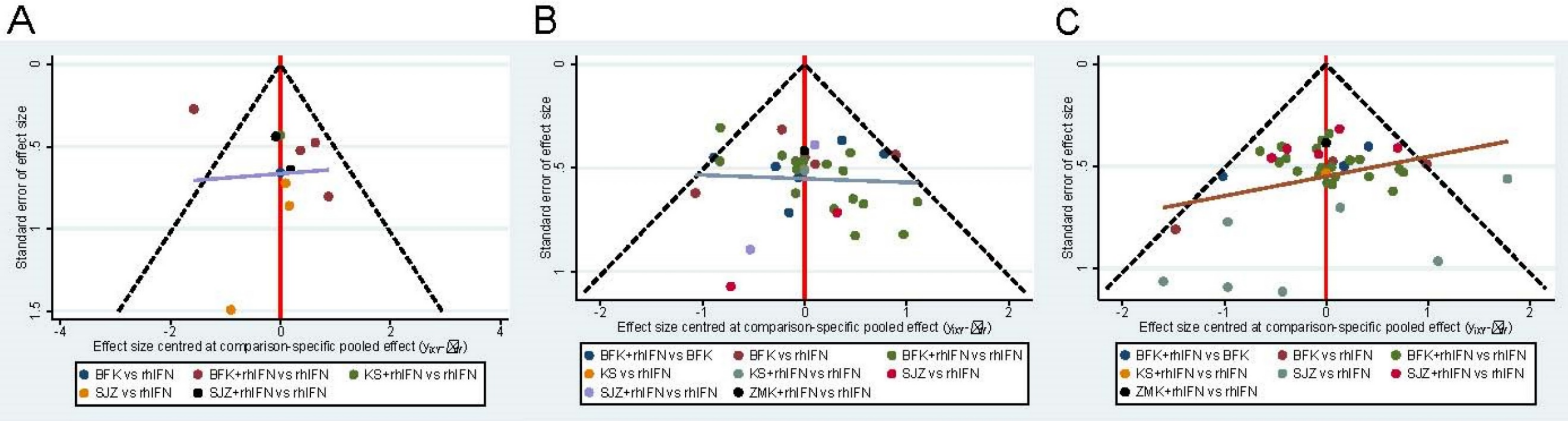
Funnel plot. BFK = Baofukang suppository; SJZ = Compound seabuckthorn seed oil suppository; KS = Kushen gel; ZMK = Zhimikang suppository; rhIFN = recombinant human interferon. **(A)** The rate of HR-HPV clearance follow-up at 6 months; **(B)** The rate of HR-HPV clearance after treatment; **(C)** The clinical effectiveness rate.

The funnel plot of the three main results shows the publication bias in **Figure 5**. All funnel diagrams show that not all studies are symmetrically distributed around the X = 0 line, and each adjusted guideline is not vertical to the centerline. Therefore, there may be significant publication biases. In addition, some of the trials are located outside the funnel plot, which provides evidence for the effect of small samples in the research network. **Supplementary S13** showed that there was no statistically significant inconsistency in the closed-loop of two outcomes (the rate of HR-HPV clearance after treatment and the clinical effectiveness rate) (*p* > 0.05), indicating that the results were reliable.

## Discussion

The results of this Bayesian network meta-analysis included 60 RCTs to assess the effects of 8 interventions on three outcomes in people with HR-HPV infection. From the results of network meta-analysis, it can be seen that BFK + rhIFN showed the best effect on long-term HR-HPV clear and KS + rhIFN had the best clinical efficacy. In addition, BFK + rhIFN did not appear to increase adverse effects. In summary, the results of this network meta-analysis have certain enlightenment significance for the clinical application and development of traditional Chinese medicine in the prevention and treatment for HR-HPV infection.

HPV is a small non-enveloped long double-stranded circular DNA virus, and the expression of viral coding genes E6 and E7 in host cells is a key factor in the occurrence of cervical cancer. Viral DNA integrates E6 and E7-based gene fragments into host cell DNA and activates E6 and E7 gene fragments that regulate virus growth, allowing them to transcribe without restriction, resulting in changes such as immortalized host cells and the evolution of malignant cells 36. CIN is characterized by cellular changes in the transformation zone of the cervix. The primary etiological factor of CIN is commonly attributed to HPV infections, particularly high-risk HPV strains such as types 16 and 18, which are responsible for over 70% of cervical cancer cases 37, 38. In addition, more and more studies 39, 40 have proposed that vaginal microecology is associated with cervical HR-HPV infection. The research discovered that women infected with HPV exhibited an augmentation in bacterial diversity in their vaginal secretions, accompanied by a reduction in the quantity of lactic acid bacteria 41. Furthermore, women with a total absence of lactic acid bacteria in their vaginal flora were twice as likely as the general population to contract HPV and develop cervical lesions. Under the dual effects of persistent HPV infection and vaginal microecological imbalance, the cervical disease gradually progresses and develops into cervical cancer.

In 2021, the World Health Organization's (WHO) “WHO guideline for screening and treatment of cervical pre-cancer lesions for cervical cancer prevention, second edition” specifically emphasized and recommended HPV-DNA testing as the preferred method for cervical cancer screening. In nations that have implemented cytology-based screening and treatment programs, the mortality rate associated with cervical cancer has decreased by a factor of five over the past 50 years 42. WHO suggests that women who are HPV-positive should all be treated. The development of cervical cancer from cervical HPV infection typically spans over a decade, and the most effective treatment time and method are still controversial. Surgical intervention and physical therapy are commonly employed to eliminate HPV infection by excising or ablating affected tissue, particularly in cases of low or high-grade cervical lesions. However, there is a possibility of residual lesions after treatment and a risk of recurrence. Drug treatment is suitable for patients with early HPV infection or young patients who have not yet given birth, with minimal harm 43. But the efficacy of HPV removal is still unknown, and there are a series of adverse reactions and complications 44. Due to the gradual younger incidence of cervical cancer, the preservation of fertility in women of reproductive age is currently a huge challenge. The majority of conventional treatments for gynecologic cancer, such as surgical procedures, gonadotoxic chemotherapy, and radiation therapy, pose a significant risk to a woman's fertility 45. Therefore, fertility preservation constitutes a crucial element in the process of clinical decision-making and treatment planning 46, 47. Women of childbearing age diagnosed with cervical cancer may seek assisted reproductive technologies to achieve their fertility wishes. At present, vitrification is a cryopreservation technique widely used in ART to preserve female fertility 48. However, considering recurrence and survival rates of cervical cancer, need to further monitored with long-term prognosis 49. Another important aspect to consider is that there was no firm conclusion on the length of follow-up for cervical cancer in the published studies 50.

In recent years, Chinese patent medicines have been widely used for cervical HR-HPV infection. According to studies conducted on cells and animals, Chinese medicine inhibits the expression of HPV16 E6 and E7 genes, regulating immune function 51. Several randomized controlled trials 9, 52, 53 have shown that Chinese patent medicines alone or in combination with recombinant human interferon can effectively increase the clearance rate of HPV, the level of inflammatory factors, reduce the degree of cervical erosion, and enhance the immunity of the patient. Meng Yao et al 54 found that traditional Chinese medicine plays an important role in regulating vaginal microecology, and its specific mechanism of action may be to reshape the local microbial community of the vagina.

For the primary outcome, BFK + rhIFN ranked first (SUCRA = 78.16%). Baofukang suppository, a Chinese medication with a long history of usage for treating gynecological illnesses, has been consistently reported in recent decades that its combination with recombinant human interferon-2b is effective for the treatment of cervical HPV infection. A meta-analysis reported that the HPV clearance rate and total effective rate of the combination of BFK and interferon were superior to those of interferon alone 35. It consists mostly of two types of Chinese medicine: zedoary turmeric oil and borneol. Zedoary turmeric oil is a volatile oil distilled from turmeric that includes antibacterial, antineoplastic, and antiviral active compounds such as curcumin, curcuminol, and curcuminone 55. Borneol is a natural crystalline chemical derived from the resin of the Dipterocarpaceae tree that contains bicyclic monoterpene alcohol, which has anti-inflammatory, analgesic, and antibacterial activities and can boost medication bioavailability in brain tissue 56. A study reported that BFK can inhibit the cervical carcinoma cell directly and also restrain the tumor by depressing the expression of HPV16/E6E7 genes 57. Through the combined application with recombinant human interferon, it can enhance phagocyte ability, the efficacy of complex infection and multi-type infection, improve the body's immunity and resistance, and the long-term effect of virus clearance is better 58, 59. Clinical research showed that traditional Chinese medicine has the potential to decrease the incidence of persistent HR-HPV infection, reduce recurrence, and prevent cervical lesions through the regulation of vaginal flora density and diversity, vaginal pH levels, and the balance of vaginal flora 60. The treatment effect of traditional Chinese medicine combined with interferon is more significant than the single vaginal use of interferon. Furthermore, the administration route and duration of treatment are generally well-tolerated by the majority of patients.

KS + rhIFN performed the best for improving the rate of HR-HPV clearance after treatment (SUCRA = 90.77%) and the clinical effectiveness rate (SUCRA = 87.39%). The main component of KS is matrine, which has antibacterial, anti-inflammatory, insecticidal, itching, antipyretic, analgesic, immunomodulation, antitumor, and other pharmacological effects 61. In addition, matrine can also enhance the activity of vaginal lactobacillus, regulate immunity, antibacterial and anti-inflammatory, which is conducive to restoring the balance of the vaginal microecology of patients and promoting the body's clearance of viruses^62^, ^63^. In recent years, it has made certain clinical and experimental research progress in the treatment of HPV infection 64-66. The main components of Zhimikang suppository are cortex phellodendri, sophora, acacia catechu, alum and borneol, which have the effects of anti-virus, anti-pathogenic microorganisms, saprophytic muscle, promoting tissue renewal and repair, and improving cervical inflammation 67. Modern pharmacological studies have shown that compound seabuckthorm seed oil suppository can effectively improve the body's immunity, block virus replication, regulate vaginal acid-base balance, especially for the growth and reproduction of HPV has a significant blocking effect. It also has antibacterial, anti-inflammatory and anti-tumor cell effects, which can effectively alleviate the development of cervicitis lesions, improve its clinical symptoms, and reduce the recurrence rate after medication 68. However, in the results of this network meta-analysis, the efficacy of compound seabuckthorn seed oil suppository combined with interferon in improving cervical symptoms is not very prominent.

Post-treatment HPV persistence estimates varied widely and were influenced by patient age, HPV type, detection method, treatment method, and minimum HPV post-treatment testing interval. Overall, HPV infection tended to gradually clear after CIN treatment for the different treatment modalities 69. According to the findings of Lianhui Liu et al. 70, Alan Yue et al. 71, and Shudan Zhang et al. 72, the highest HPV conversion rate occurred 6 months after the end of medication, and the lowest conversion rate after 1 year. According to the current study, after the clinician fully evaluates the patient, the observation time of stopping the drug can be divided six months after the end of medication. This follow-up time may help alleviate patient anxiety and promote autoimmune clearance, ultimately reducing medical waste.

Of the included literatures, only BFK, rhIFN, and BFK + rhIFN mentioned the occurrence of adverse events. Most adverse reactions are related to the sensitivity of the cervical tissue caused by the patient's hormone levels, which can disappear on their own without serious reactions such as local infection and cervical injury. Due to the limitations of the literature, it is not yet possible to compare the safety of proprietary Chinese medicines, and more trials observing the safety indicators of proprietary Chinese medicines will be needed in the future to verify this.

The combination of Chinese patent medicines and recombinant human interferon performs well in clinical efficiency and HR-HPV clearance rate, thereby providing guidance for the clinical treatment of HR-HPV infection. For patients with a long-term infection and weakened immunity, a combination of BFK and recombinant human interferon may be considered. If cervical symptoms are evident, KS combined with recombinant human interferon may be considered. However, these results must be interpreted cautiously.

### The Innovations and Limitations of this Study

The first network meta-analysis of different Chinese patent medicines for cervical HR-HPV infection was conducted to provide evidence to support and create traditional Chinese medicine guideline recommendations. We conducted detailed literature reviews and screening, and independently extracted data to ensure that our results were comprehensive and rigorous based on the same criteria. We rigorously conducted subgroup analyses with different time nodes to explore the efficacy of proprietary Chinese medicines at different detection time nodes.

However, our network meta-analysis has some limitations. First, all included trials were at high or unclear risk of bias, making the quality of evidence low or very low across all comparisons. Second, the duration of HR-HPV infection, the evaluation criteria for clinical efficacy vary greatly in different trials, and some studies did not mention more details. We recognized that there would be heterogeneity in definitions between trials. Third, there will be some limitations on the external adaptability of the findings, as all the studies were conducted in China. In view of the imperfect design scheme of the included literature, it is recommended that forthcoming randomized controlled trials provide comprehensive descriptions of randomization, allocation concealment, and blinding. Furthermore, the correlation between the duration of treatment of Chinese patent medicines, the follow-up time after treatment, and the rate of negative conversion is a clinically significant matter, and future research can increase the reports in this regard.

## Conclusion

Chinese patent medicines combined with rhIFN can be more effective than rhIFN alone in clearing the virus and improving cervical symptoms. In terms of clinical efficacy, BFK + rhIFN and KS + rhIFN may be the best treatments for cervical HR-HPV infection. However, due to the poor methodological quality of the included trials, our conclusions should be carefully interpreted. More high-quality randomized controlled trials are required in the future to confirm the efficacy and safety of proprietary Chinese medicines for cervical HR-HPV infection.

## Supplementary Material

Supplementary information and tables.

## Figures and Tables

**Table 1:**
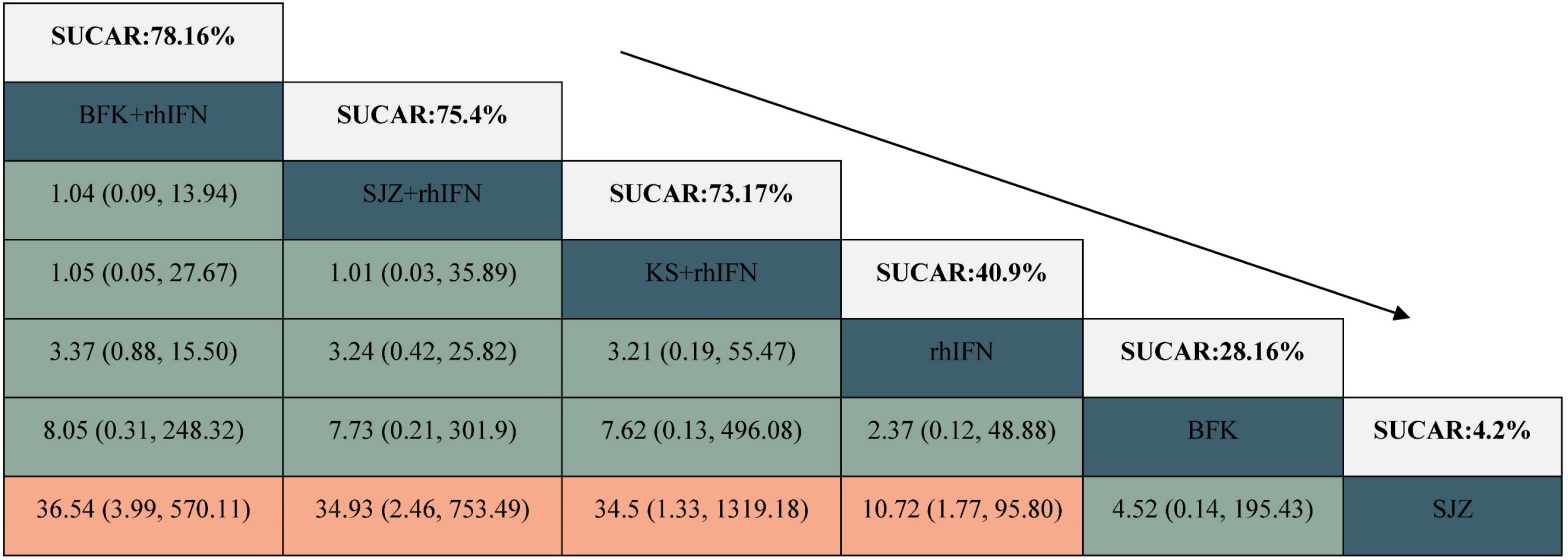
League table for the rate of HR-HPV clearance follow-up at 6 months.

BFK = Baofukang suppository; SJZ = Compound seabuckthorn seed oil suppository; KS = Kushen gel; ZMK = Zhimikang suppository; rhIFN = recombinant human interferon. SUCRA = surface under the cumulative ranking.

**Table 2:**
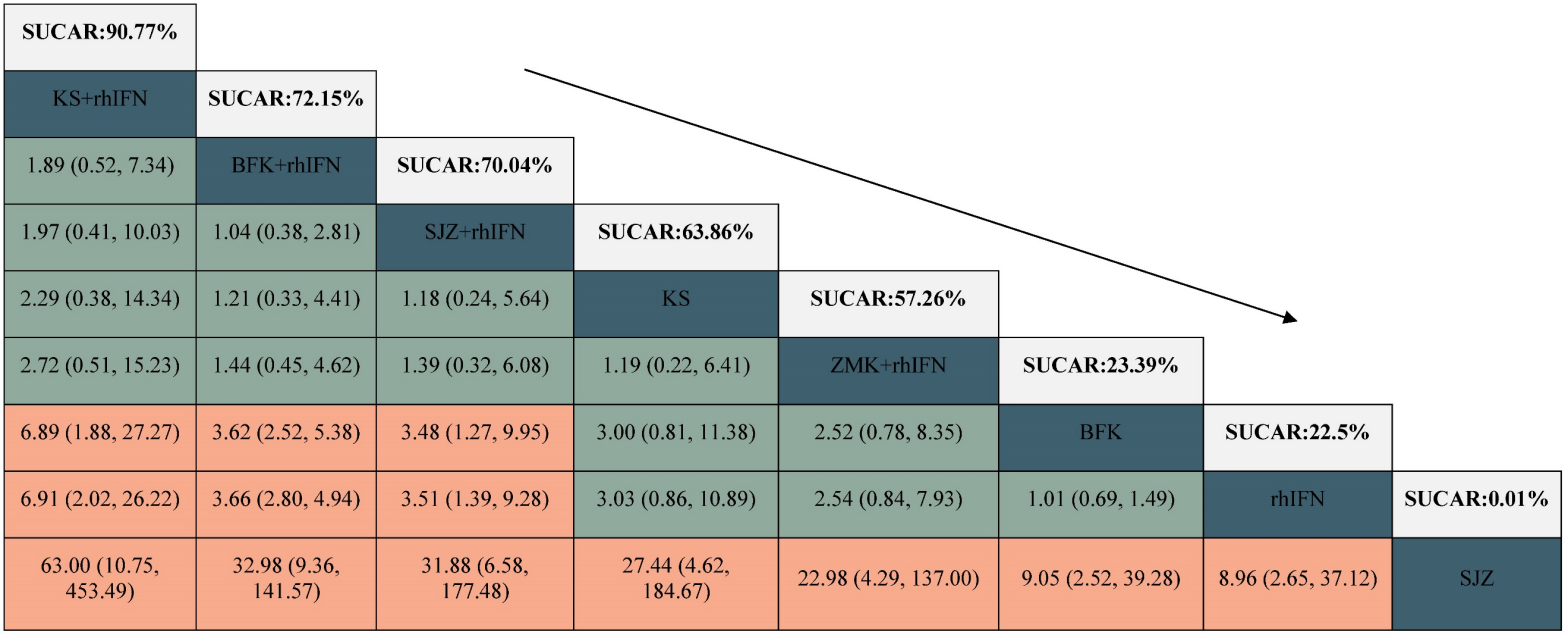
League table for the rate of HR-HPV clearance after treatment.

BFK = Baofukang suppository; SJZ = Compound seabuckthorn seed oil suppository; KS = Kushen gel; ZMK = Zhimikang suppository; rhIFN = recombinant human interferon. SUCRA = surface under the cumulative ranking.

**Table 3:**
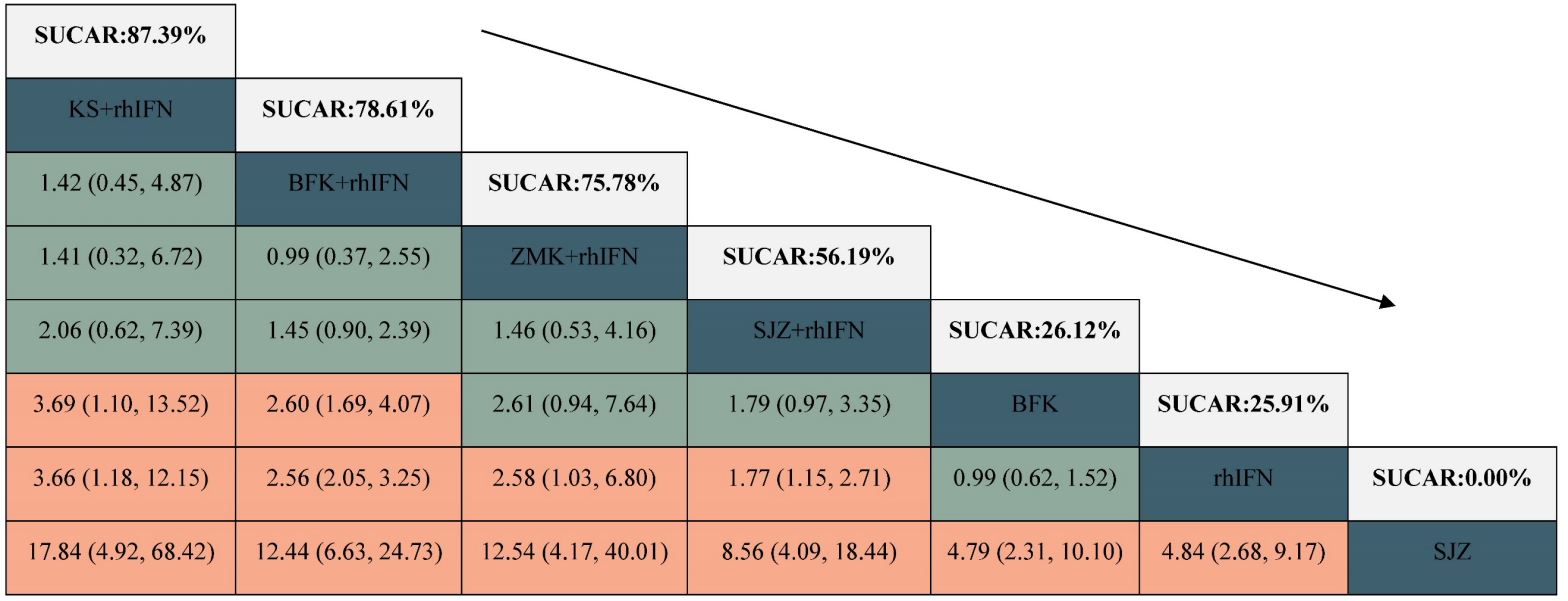
League table for the clinical effectiveness rate.

BFK = Baofukang suppository; SJZ = Compound seabuckthorn seed oil suppository; KS = Kushen gel; ZMK = Zhimikang suppository; rhIFN = recombinant human interferon. SUCRA = surface under the cumulative ranking.

## Abbreviations

HR-HPV: high-risk human papillomavirus
CIN: cervical intraepithelial neoplasia
FIGO: International Federation of Gynecology and Obstetrics
BFK: Baofukang suppository
KS: Kushen gel
NMA: network meta-analysis
PRISMA: Preferred Reporting Items for Systematic reviews and Meta-Analyses
PROSPERO: International Prospective Registration of Systematic Reviews
RCT: randomized controlled trial
CBM: Chinese Biomedical Databases
CNKI: China National Knowledge Infrastructure
VIP: Chinese Scientific Journals Database
MeSH: Medical Subject Heading
ASC-US: cytology-atypical squamous cells of undetermined significance
ASCCP: American Society for Colposcopy and Cervical Pathology
ACOG: American College of Obstetricians and Gynecologists
CSCCP: Chinese Society for Colposcopy and Cervical Pathology of China Healthy Birth Science Association
GRADE: grading of recommendations assessment, development, and evaluation
DIC: deviance information criterion
OR: odds ratio
CrI: credible intervals
SUCRA: surface under the cumulative ranking curve
World Health Organization: WHO
rhIFN: recombinant human interferon
SJZ: Compound seabuckthorn seed oil suppository
ZMK: Zhimikang suppository
