# Supplementary material for: Comparative Efficacy and Safety of Chinese Patent Medicines for Cervical High-risk Human Papillomavirus Infection: A Bayesian Network Meta-analysis
Source: J Cancer. 2023 Jul 31;14(12):2373–85. doi: 10.7150/jca.86043 (PMC10414044; doi:10.7150/jca.86043)

**Supplementary S1. PRISMA NMA Checklist of Items to Include When Reporting A Systematic Review Involving a Network Meta-analysis**

| Section/Topic | Item # | Checklist Item | Reported on Page # |
|---|---|---|---|
| **TITLE** | | | |
| Title | 1 | Identify the report as a systematic review *incorporating a network meta-analysis (or related form of meta-analysis).* | **Page. 1** |
| **ABSTRACT** | | | |
| Structured summary | 2 | Provide a structured summary including, as applicable:<br>**Background:** main objectives<br>**Methods:** data sources; study eligibility criteria, participants, and interventions; study appraisal; and *synthesis methods, such as network meta-analysis.*<br>**Results:** number of studies and participants identified; summary estimates with corresponding confidence/credible intervals; *treatment rankings may also be discussed. Authors may choose to summarize pairwise comparisons against a chosen treatment included in their analyses for brevity.*<br>**Discussion/Conclusions:** limitations; conclusions and implications of findings.<br>**Other:** primary source of funding; systematic review registration number with registry name. | **Page. 1** |
| **INTRODUCTION** | | | |
| Rationale | 3 | Describe the rationale for the review in the context of what is already known, *including mention of why a network meta-analysis has been conducted.* | **Page. 1-2** |
| Objectives | 4 | Provide an explicit statement of questions being addressed, with reference to participants, interventions, comparisons, outcomes, and study design (PICOS). | **Page. 1-2** |
| **METHODS** | | | |
| Protocol and registration | 5 | Indicate whether a review protocol exists and if and where it can be accessed (e.g., Web address); and, if available, provide registration information, including registration number. | **Page. 2** |
| Eligibility criteria | 6 | Specify study characteristics (e.g., PICOS, length of follow-up) and report characteristics (e.g., years considered, language, publication status) used as criteria for eligibility, giving rationale. *Clearly describe eligible treatments included in the treatment network, and note whether any have been clustered or merged into the same node (with justification).* | **Page. 2-4** |
| Information sources | 7 | Describe all information sources (e.g., databases with dates of coverage, contact with study authors to identify additional studies) in the search and date last searched. | **Page. 3** |
| Search | 8 | Present full electronic search strategy for at least one database, including any limits used, such that it could be repeated. | **Supplementary S2** |
| Study selection | 9 | State the process for selecting studies (i.e., screening, eligibility, included in systematic review, and, if applicable, | **Page. 3-5** |

| | | | |
|---|---|---|---|
| | | included in the meta-analysis). | |
| Data collection process | 10 | Describe method of data extraction from reports (e.g., piloted forms, independently, in duplicate) and any processes for obtaining and confirming data from investigators. | **Page. 3-4** |
| Data items | 11 | List and define all variables for which data were sought (e.g., PICOS, funding sources) and any assumptions and simplifications made. | **Page. 3-4** |
| **Geometry of the network** | **S1** | Describe methods used to explore the geometry of the treatment network under study and potential biases related to it. This should include how the evidence base has been graphically summarized for presentation, and what characteristics were compiled and used to describe the evidence base to readers. | **Page. 4-5** |
| Risk of bias within individual studies | 12 | Describe methods used for assessing risk of bias of individual studies (including specification of whether this was done at the study or outcome level), and how this information is to be used in any data synthesis. | **Page. 4-5** |
| Summary measures | 13 | State the principal summary measures (e.g., risk ratio, difference in means). *Also describe the use of additional summary measures assessed, such as treatment rankings and surface under the cumulative ranking curve (SUCRA) values, as well as modified approaches used to present summary findings from meta-analyses.* | **Page. 4-5** |
| Planned methods of analysis | 14 | Describe the methods of handling data and combining results of studies for each network meta-analysis. This should include, but not be limited to:<br>• *Handling of multi-arm trials;*<br>• *Selection of variance structure;*<br>• *Selection of prior distributions in Bayesian analyses; and*<br>• *Assessment of model fit.* | **Page. 4-5** |
| **Assessment of Inconsistency** | **S2** | Describe the statistical methods used to evaluate the agreement of direct and indirect evidence in the treatment network(s) studied. Describe efforts taken to address its presence when found. | **Page. 4-5** |
| Risk of bias across studies | 15 | Specify any assessment of risk of bias that may affect the cumulative evidence (e.g., publication bias, selective reporting within studies). | **Page. 5 Supplementary S6** |
| Additional analyses | 16 | Describe methods of additional analyses if done, indicating which were pre-specified. This may include, but not be limited to, the following:<br>• Sensitivity or subgroup analyses;<br>• Meta-regression analyses;<br>• *Alternative formulations of the treatment network; and*<br>• *Use of alternative prior distributions for Bayesian analyses (if applicable).* | |

**RESULTS†**

| | | | |
|---|---|---|---|
| Study selection | 17 | Give numbers of studies screened, assessed for eligibility, and included in the review, with reasons for exclusions at each stage, ideally with a flow diagram. | **Page. 5 and Figure. 1** |
| **Presentation of network structure** | **S3** | Provide a network graph of the included studies to enable visualization of the geometry of the treatment network. | **Figure. 2** |
| **Summary of network geometry** | **S4** | Provide a brief overview of characteristics of the treatment network. This may include commentary on the abundance of trials and randomized patients for the different interventions and pairwise comparisons in the network, gaps of evidence in the treatment network, and potential biases reflected by the network structure. | **Page. 5 and Figure. 2** |
| Study characteristics | 18 | For each study, present characteristics for which data were extracted (e.g., study size, PICOS, follow-up period) and provide the citations. | **Page. 5 and Supplementary S4** |
| Risk of bias within studies | 19 | Present data on risk of bias of each study and, if available, any outcome level assessment. | **Page.5 and Supplementary S5** |
| Results of individual studies | 20 | For all outcomes considered (benefits or harms), present, for each study: 1) simple summary data for each intervention group, and 2) effect estimates and confidence intervals. *Modified approaches may be needed to deal with information from larger networks.* | **Supplementary S10** |
| Synthesis of results | 21 | Present results of each meta-analysis done, including confidence/credible intervals. *In larger networks, authors may focus on comparisons versus a particular comparator (e.g. placebo or standard care), with full findings presented in an appendix. League tables and forest plots may be considered to summarize pairwise comparisons.* If additional summary measures were explored (such as treatment rankings), these should also be presented. | **Page. 5-6 and Table. 1-3 and Supplementary S11** |
| **Exploration for inconsistency** | **S5** | Describe results from investigations of inconsistency. This may include such information as measures of model fit to compare consistency and inconsistency models, *P* values from statistical tests, or summary of inconsistency estimates from different parts of the treatment network. | **Page. 6 and Supplementary S13** |
| Risk of bias across studies | 22 | Present results of any assessment of risk of bias across studies for the evidence base being studied. | **Supplementary S6-S9** |
| Results of additional analyses | 23 | Give results of additional analyses, if done (e.g., sensitivity or subgroup analyses, meta-regression analyses, *alternative network geometries studied, alternative choice of prior distributions for Bayesian analyses,* and so forth). | **Page. 4-6** |

**DISCUSSION**

| | | | |
|---|---|---|---|
| Summary of evidence | 24 | Summarize the main findings, including the strength of evidence for each main outcome; consider their relevance to key groups (e.g., healthcare providers, users, and policy-makers). | **Page. 7-9** |
| Limitations | 25 | Discuss limitations at study and outcome level (e.g., risk of bias), and at review level (e.g., incomplete retrieval of identified research, reporting bias). *Comment on the validity of the assumptions, such as transitivity and consistency. Comment* | **Page. 9** |

| | | | |
|---|---|---|---|
| | | *on any concerns regarding network geometry (e.g., avoidance of certain comparisons).* | |
| Conclusions | 26 | Provide a general interpretation of the results in the context of other evidence, and implications for future research. | **Page. 9** |
| Funding | 27 | Describe sources of funding for the systematic review and other support (e.g., supply of data); role of funders for the systematic review. This should also include information regarding whether funding has been received from manufacturers of treatments in the network and/or whether some of the authors are content experts with professional conflicts of interest that could affect | **Page. 9** |

PICOS = population, intervention, comparators, outcomes, study design.
* Text in italics indicateS wording specific to reporting of network meta-analyses that has been added to guidance from the PRISMA statement.

† Authors may wish to plan for use of appendices to present all relevant information in full detail for items in this section.

**Supplementary S2: Search strategy**

**PubMed (August 2022)**

| Number | Search Terms | Number of Citations |
|---|---|---|
| 1 | papillomavirus infections [MeSH Terms] | 33,848 |
| 2 | ((((((((((((Papillomavirus Infection[Title/Abstract]) OR (Human Papillomavirus Infection[Title/Abstract])) OR (Human Papillomavirus Infections[Title/Abstract])) OR (Papillomavirus Infection, Human[Title/Abstract])) OR (Papillomavirus Infections, Human[Title/Abstract])) OR (HPV Infection[Title/Abstract])) OR (HPV Infections[Title/Abstract])) OR (high-risk human papillomavirus infection[Title/Abstract])) OR (high-risk HPV infection[Title/Abstract])) OR (cervical intraepithelial neoplasia[Title/Abstract])) OR (hr-HPV[Title/Abstract])) OR (ASC-US[Title/Abstract])) OR (LSIL[Title/Abstract])) OR (CIN1[Title/Abstract]) | 25,719 |
| 3 | 1 or 2 | 49,674 |
| 4 | Medicine, Chinese Traditional [MeSH Terms] | 22,883 |
| 5 | (((((Chinese herbal[Title/Abstract]) OR (Traditional Chinese Medicine[Title/Abstract])) OR (Chinese patent medicine[Title/Abstract])) OR (Chinese and Western Medicine[Title/Abstract])) OR (Integrated Chinese and Western Medicine[Title/Abstract])) OR (suppository[Title/Abstract]) | 39,983 |
| 6 | 4 or 5 | 52,175 |
| 7 | **3 and 6** | **32** |

**Embase (August 2022)**

| Number | Search Terms | Number of Citations |
|---|---|---|
| 1 | MeSH descriptor: [Papillomavirus Infections] explode all trees | 1,845 |
| 2 | ("Papillomavirus Infection" OR "Human Papillomavirus Infection" OR "Human Papillomavirus Infections" OR "Papillomavirus Infection, Human" OR "Papillomavirus Infections, Human" OR " HPV Infection" OR "HPV Infections" OR "high-risk human papillomavirus infection" OR "high-risk HPV infection" OR "cervical intraepithelial neoplasia" OR "hr-HPV" OR "ASC-US" OR "LSIL" OR "CIN1"):ti,ab,kw | 2,577 |
| 3 | 1 or 2 | 3,563 |
| 4 | MeSH descriptor: [Medicine, Chinese Traditional] explode all trees | 1,549 |
| 5 | ("Chinese herbal" OR "Traditional Chinese Medicine" OR "Chinese patent medicine" OR "Chinese and Western Medicine" | 14,452 |

| | | |
|---|---|---|
| | OR "Integrated Chinese and Western Medicine" OR "suppository"):ti,ab,kw | |
| 6 | 4 or 5 | 16,743 |
| 7 | **3 and 6** | **39** |

**Cochrane Central Register of Controlled Trials (August 2022)**

| Number | Search Terms | Number of Citations |
|---|---|---|
| 1 | 'papillomavirus infection'/exp | 1,845 |
| 2 | 'Papillomavirus Infection':ab,ti OR 'Human Papillomavirus Infection':ab,ti OR 'Human Papillomavirus Infections':ab,ti OR 'Papillomavirus Infection, Human':ab,ti OR 'HPV Infection':ab,ti OR 'HPV Infections':ab,ti OR 'high-risk human papillomavirus infection':ab,ti OR 'high-risk HPV infection':ab,ti OR 'cervical intraepithelial neoplasia':ab,ti OR 'hr-HPV':ab,ti OR 'ASC-US':ab,ti OR 'LSIL':ab,ti OR 'CIN1':ab,ti | 1,916 |
| 3 | 1 or 2 | 3,787 |
| 4 | 'Medicine, Chinese Traditional'/exp | 8,061 |
| 5 | 'Chinese herbal':ab,ti OR 'Traditional Chinese Medicine':ab,ti OR 'Chinese patent medicine':ab,ti OR 'Chinese and Western Medicine':ab,ti OR 'Integrated Chinese and Western Medicine':ab,ti OR 'suppository':ab,ti | 6,654 |
| 6 | 4 or 5 | 10,275 |
| 7 | **3 and 6** | **16** |

**Web of Science (August 2022)**

| Number | Search Terms | Number of Citations |
|---|---|---|
| 1 | TS=(Papillomavirus Infection OR Human Papillomavirus Infection OR Human Papillomavirus Infections OR Papillomavirus Infection, Human OR HPV Infection OR HPV Infections OR high-risk human papillomavirus infection OR high-risk HPV infection OR cervical intraepithelial neoplasia OR hr-HPV OR ASC-US OR LSIL OR CIN1) | 67,076 |
| 2 | TS=(Chinese herbal OR Traditional Chinese Medicine OR Chinese patent medicine OR Chinese and Western Medicine OR Integrated Chinese and Western Medicine OR suppository) | 124,976 |
| 3 | **1 and 2** | **120** |

**Supplementary S3: Reference list of the included RCTs**

[1]Tang M. Y., Tang D. Y., Li J. F. (2018).A clinical study on treating high-risk HPV persistent infection with the Baofukang suppository plus rhIFN. Clinical Journal of Chinese Medicine. (04),72-74.

[2]Wang Y. J. (2019). Effects of recombinant human interferon-α2a suppository combined with Baofukang suppository in the treatment of cervical high-risk human papillomavirus infection on HPV DNA load and negative rate. Chinese Journal of Clinical Rational Drug Use. (16),87-89. doi:10.15887/j.cnki.13-1389/r.2019.16.048.

[3]Wang L., Zhou H. J. (2021). Efficacy of Recombinant Human Interferon Combined with Baofukang Suppositories in the Treatment of High-Risk Cervical HPV Infection. Chinese Journal of Hemorheology. 31(1):90-93. DOI:10.3969/j.issn.1009-881X.2021.01.021.

[4]Wang Y. P., Xu J., Li Y., et.al. (2021). Effect of Baofukang suppository combined with interferon suppository on high-risk human papillomavirus infection patients and changes in HPV-DNA, inflammatory factors and immune function. Guizhou Medical Journal. (01),39-40.

[5]Wu Y. M. (2015). Efficacy observation of recombinant interferon α-2b suppository combined with Baofukang suppository in the treatment of cervical high-risk papillomavirus subclinical infection. New Chinese Medicine. (09),145-146. doi:10.13457/j.cnki.jncm.2015.09.068.

[6]Wu C. M., Zhang A. Y. (2019). Clinical study of Baofukang suppository combined with recombinant human interferon α-2b in the treatment of chronic cervicitis complicated with high-risk HPV infection. Journal of Hubei University of Chinese Medicine. (06),69-71.

[7]Xiao L. L., Deng J. M. (2020). Efficacy observation of recombinant human interferon a-2b effervescent tablet combined with Baofukang suppository in the treatment of cervical high-risk human papillomavirus infection. Bao Jian Wen Hui. (6):58-59,67. DOI:10.3969/j.issn.1671-5217.2020.06.030.

[8]Yang J. L., Huang J.P. (2016). Efficacy analysis of Baofukang suppository combined with Xinfuning in cervical high-risk human papillomavirus infection. Modern Diagnosis and Treatment. (15),2810-2811.

[9]Zhu K. X. (2018). Observation on the effect of recombinant human interferon α-2a suppository combined with Baofukang suppository in the treatment of cervical high-risk HPV infection. Henan Medical Research. (04),723-724.

[10]Zhu L. F., Xu Y. (2021). Efficacy of Baofukang suppository combined with interferon α-2b in the treatment of chronic cervicitis complicated with human papillomavirus infection. Journal of Navy Medicine. (03),343-346.

[11]Chen Y. Q. (2021). Analysis of the effect of recombinant human interferon α-2b suppository combined with Baofukang suppository in the treatment of patients with chronic cervicitis and high-risk HPV infection. Strait Pharmaceutical Journal. (05),155-156.

[12]Chen C. Y., Huang R. S. (2022). Analysis of Clinical Efficacy of Interferon Combined with Baofukang Suppository in the Treatment of HR-HPV Infection. China & Foreign Medical Treatment. (13),86-89+97. doi:10.16662/j.cnki.1674-0742.2022.13.086.

[13] Chen J. (2020). Analysis of the application of Baofukang suppository combined with recombinant human interferon α-2b gel in the treatment of chronic cervicitis with high-risk HPV (HR-HPV) infection. Oriental medicinal diet. (11):42.

[14]Gao L. X. (2018). Clinical observation on chronic cervicitis combined with high-risk human papillomavirus infection treated by integrated traditional Chinese and Western medicine. Journal of Shanxi University of Chinese Medicine. (02),54-55.

[15]Geng C. C. (2020). Efficacy observation of recombinant human interferon α-2a combined with Baofukang suppository in the treatment of cervical high-risk HPV infection. Drug Evaluation. (08),27-29.

[16]Han Y. Q., Zhang L. L., Dai Q. L., et.al. (2019). Clinical study of recombinant human interferon α-2b combined with Baofukang suppository in the treatment of chronic cervicitis complicated with high-risk HPV infection. Journal of North Sichuan Medical College. (03),422-424.
[17]He Y. (2019). Observation on the effect of Baofukang suppository combined with recombinant human interferon α-2b gel in the treatment of chronic cervicitis with high-risk HPV infection. Medical Journal of Chinese People's Health. (09),49-50.
[18]Lai Z. F., Guo C., Xiao S. J., et.al. (2021). Clinical efficacy and safety of recombinant human interferon α2b suppository combined with Baofukang suppository in the treatment of cervical high-risk human papillomavirus infection. Chinese Journal of Clinical Rational Drug Use. (21),135-137. doi:10.15887/j.cnki.13-1389/r.2021.21.049.
[19]Lai X., Zhang F. Z., Liang H. M. (2022). Observation on the effect of combined application of Baofukang suppository and recombinant human interferon α-2b in the treatment of cervical high-risk HPV infection. Women's Health Research. (1):44-45.
[20]Li C. Y. (2018). Application of recombinant human interferon α2b combined with Baofukang suppository in the treatment of cervical high-risk HPV infection. Women's Health Research. (24),39+41.
[21]Li X. Y. (2020). Effect of recombinant human interferon α-2b suppository combined with Baofukang suppository in the treatment of patients with high-risk human papillomavirus infection. Medical Journal of Chinese People's Health. (05),52-54.
[22]Li H. T. (2021). Clinical efficacy of Baofukang suppository combined with recombinant human interferon α-2b in the treatment of chronic cervicitis complicated with high-risk HPV infection. Chinese Journal of Clinical Rational Drug Use. (06),49-50+53. doi:10.15887/j.cnki.13-1389/r.2021.06.017.
[23]Li R. Q., Gong Y., Ai Z. X. (2014). Curative effect observation of Baofukang suppository combined with interferon α-2b suppository in the treatment of cervical human papillomavirus subclinical infection. Jilin Medical Journal. (15),3275.
[24]Li Z. F., Ma J. H. (2018). Efficacy analysis of Baofukang suppository combined with Xinfuning in cervical high-risk human papillomavirus infection. Women's Health Research. (12),62-63.
[25]Liang N. K., Liu X. L., Lu J. W., et.al. (2019). Efficacy analysis of Baofukang suppository combined with Xinfuning in the treatment of cervical high-risk human papillomavirus infection. Electronic Journal of Practical Gynecological Endocrinology. (06),44+46. doi:10.16484/j.cnki.issn2095-8803.2019.06.027.
[26]Liu J., Qi Z. Y., He Y. J., et.al. (2016). Discussion on the treatment of cervical high-risk HPV infection. Hebei Medicine. (12),2115-2118.
[27]Liu H. M., Huang X. Y., Liu X. Y. (2016). Clinical effect of human recombinant interferon-alpha 2b gel combined with Baofukang suppository in the treatment of cervical high-risk HPV infection. Journal of Guangdong Medical University. (03),312-313.
[28]Ma Y., Liang C. P. (2021). Clinical observation of Baofukang suppository combined with recombinant human interferon α-2b gel in the treatment of cervical columnar epithelial heterotopic complicated with high-risk human papillomavirus infection. China's Naturopathy. (03),90-92. doi:10.19621/j.cnki.11-3555/r.2021.0334.
[29]Mai B., Hu G. Y., Liu T. Y., et.al. (2018). Efficacy of interferon combined with Baofukang suppository in the treatment of cervical high-risk HPV infection and its effect on vaginal microecological environment. China Practical Medicine. (36),141-143. doi:10.14163/j.cnki.11-5547/r.2018.36.076.
[30]Song X. X., Liu Y. L., Hao Q. Y. (2011). Xinfuning Combined with Baofukang Suppository for 53 Cases with Cervical High-risk Human Papillomavirus Infection. Journal of Chinese Oncology. (11),825-827.

[31]Su X. Y., Meng L. P., Zou C. C., et.al. (2020). Clinical Observation of Recombinant Human Interferon Gel Combined with Baofukang Suppository in the Treatment of Cervical High-risk HPV Infection. China Pharmacy. (08),984-988.
[32]Cao Y. M. (2017). Efficacy observation of interferon α-2b suppository combined with compound seabuckthorn seed oil suppository in the treatment of chronic cervicitis with high-risk papillomavirus infection. Modern Journal of Integrated Traditional Chinese and Western Medicine. (20),2246-2248.
[33]Han F. Q. (2020). Effects of recombinant human interferon α-2b gel combined with compound seabuckthorn seed oil suppository in treatment of chronic cervicitis with high-risk HPV infection. Medical Journal of Chinese People's Health. (23),19-20+23.
[34]Hu Y. Y. (2018). Efficacy analysis of compound seabuckthorn seed oil suppository combined with recombinant human interferon in the treatment of high-risk HPV infection complicated with chronic cervicitis. Modern Diagnosis and Treatment. (14),2263-2264.
[35]Shen J. H. (2015). Clinical observation of interferon α-2b suppository combined with compound seabuckthorn seed oil suppository in the treatment of chronic cervicitis with high-risk papillomavirus infection. New Chinese Medicine. (11),133-134. doi:10.13457/j.cnki.jncm.2015.11.061.
[36] Wu C. Y., Feng Y. Y. (2018). Clinical effect of recombinant human interferon α-2b gel combined with compound sea-buckthorn seed oil suppository in the treatment of chronic cervicitis with high-risk type HPV infection. Clinical Research and Practice. (19):134-135. DOI:10.19347/j.cnki.2096-1413.201819065.
[37]Pan W. J., Zhang L. N. (2021). Effect of Kushen gel Combined with interferon Suppositories on Negative Conversion rate and curative effect of Cervicitis patients with high-risk HPV infection. Electronic Journal of Practical Gynecological Endocrinology. (09),71-73. doi:10.16484/j.cnki.issn2095-8803.2021.09.007.
[38]Zhao H. D., Feng X. L., Zhao Y. (2016). Randomized controlled study: Sophora flavescens gel in treatment of cervical HPV infection. China Journal of Chinese Materia Medica. (21),4072-4075.
[39]Chen D. Y. (2021). Efficacy analysis of Zhimikang suppository combined with recombinant human interferon-α2b suppository in patients with high-risk human papillomavirus-infected chronic cervicitis. Anti-Infection Pharmacy. (03),459-462. doi:10.13493/j.issn.1672-7878.2021.03-043.
[40]Du.X. X. (2020). Efficacy of recombinant human interferon α-2b in the treatment of cervical lesions with high-risk HPV infection by combination with maternal suppositories. Practical Clinical Journal of Integrated Traditional Chinese and Western Medicine. (14),31-32. doi:10.13638/j.issn.1671-4040.2020.14.015.
[41]Hu. J. Y. (2017). Observation of the effect of maternal health suppositories combined with recombinant human interferon suppositories in the treatment of high-risk human papillomavirus persistent infection. Jiangsu Journal of Preventive Medicine. (06),704-705. doi:10.13668/j.issn.1006-9070.2017.06.36.
[42]Ma. L. (2019). Efficacy of recombinant human interferon α-2b gel in combination with maternal suppositories in the treatment of high-risk HPV infection of the cervix and its effect on immune factors associated with the microecological environment. Proceeding of Clinical Medicine. (12),886-889. doi:10.16047/j.cnki.cn14-1300/r.2019.12.002.
[43]Sun. D. D. (2021). Observation of the effect of the combination of recombinant human interferon α-2b in the treatment of chronic cervicitis and high-risk human papillomavirus infection. Chinese Journal of Modern Drug Application. (18),139-141. doi:10.14164/j.cnki.cn11-5581/r.2021.18.052.
[44]Xu. H. L., Nie. J. (2022). Clinical efficacy of recombinant human interferon alfa-2b in combination with maternal suppositories in patients with chronic cervicitis and high-risk HPV infection. Chinese Journal of Clinical Rational Drug Use. (15),143-146. doi:10.15887/j.cnki.13-1389/r.2022.15.045.

[45]Yin. S. X. (2019). Clinical effect of cervical lesions with high-risk HPV infection with recombinant human interferon α-2b vaginal effervescent capsules. Chinese Journal of Modern Drug Application. (17),3-5. doi:10.14164/j.cnki.cn11-5581/r.2019.17.002.
[46]Li. D. M. (2021). Efficacy of interferon in the treatment of high-risk HPV infection in the treatment of maternal suppository and the evaluation of the impact on the vaginal microecological environment. Doctor. 6(6):113-114.
[47]Zhang. J. J. (2016). Discussion on the clinical efficacy of interferon suppositories in the treatment of high-risk HPV infection with maternal health suppositories. China Continuing Medical Education. (17),218-219.
[48]Ye. Y. L., Sang X. Q., Xiong F., et.al. (2015). Clinical observation of traditional Chinese medicine combined with maternal health suppositories in the treatment of cervical high-risk HPV infection and CIN I. Zhejiang Journal of Traditional Chinese Medicine. (07),478-479. doi:10.13633/j.cnki.zjtcm.2015.07.006.
[49]Xu. Y. H. (2016). Efficacy of maternal health suppositories and interferon alfa-2b suppositories in the treatment of high-risk cervical HPV persistent infection. Henan Medical Research. (09),1634-1635.
[50]Zhang Y. P. (2017). Clinical efficacy analysis of You Jing'an in the treatment of chronic cervicitis complicated by high-risk HPV infection in 40 cases. The Medical Forum. (20),2614-2615. doi:10.19435/j.1672-1721.2017.20.013.
[51]Wu. Y. J., Sun. F. L., Hu. Y. H., et.al. (2018). Efficacy analysis of maternal health suppositories in the treatment of high-risk HPV infection of the cervix. Heilongjiang Medicine and Pharmacy. (02),166-167.
[52]Zhang. D. W. (2019). Effect of recombinant human interferon α-2b gel on clinical efficacy and safety in patients with CIN1 and high-risk HPV infection. Modern Medicine and Health Research Electronic Journal. (07),116-117.
[53]Huang. W. L., Chen. Q. (2012). Efficacy of recombinant human interferon α-2b gel in the treatment of chronic cervicitis complicated by high-risk HPV infection. Hainan Medical Journal. (05),28-30.
[54]Huang. L., Wu. L. L., Jiang. Y. (2013). Recombinant human interferon for chronic cervicitis and high-risk HPV infection in 30 cases. China Pharmaceuticals. (11),91-92.
[55]Li. F. (2017). Analysis of the therapeutic value of recombinant human interferon in chronic cervicitis and high-risk human papillomavirus infection. Guide of China Medicine. (13),75-76. doi:10.15912/j.cnki.gocm.2017.13.055.
[56]Liu. D. M. (2017). Clinical efficacy of recombinant human interferon α-2b gel in the treatment of chronic cervicitis complicated by high-risk HPV infection. Women's Health Research. (20),96-97.
[57]Qiu. C. H. (2017). Clinical efficacy of recombinant human interferon α-2b gel in the treatment of chronic cervicitis complicated by high-risk human papillomavirus infection. China Modern Medicine. (07),127-129.
[58]Du. L. L. (2018). Recombinant human interferon α-2b gel for chronic cervicitis with high-risk HPV infection of clinical effects. Doctor. (Z2),121-122. doi:10.19604/j.cnki.dys.2018.z2.147.
[59]Hua. W. (2019). Clinical value and experience of recombinant human interferon α-2b gel in the treatment of chronic cervicitis complicated by high-risk human papillomavirus infection. Guide of China Medicine. (15),53-54. doi:10.15912/j.cnki.gocm.2019.15.029.
[60]Zhang. Y. Z. (2019). To analyze the feasibility of recombinant human interferon α2a suppositional adjuvant therapy for chronic cervicitis complicated by high-risk human papillomavirus (HPV) infection. World Latest Medicine Information. (88),156-157. doi:10.19613/j.cnki.1671-3141.2019.88.102.

**Supplementary S4: Basic features of the included studies**

| No. | Study ID | Sample size/ Experiment/Control | Age(years, $\overline{x}$±s) | | Duration of disease($\overline{x}$±s) | | Intervention | | Intervention duration | Outcomes |
|---|---|---|---|---|---|---|---|---|---|---|
| | | | Experiment | Control | Experiment | Control | Experiment | Control | | |
| 1 | Tang and Tang et al. (2018) | 20/20 | 52.49 ± 4.60 | 53.02 ± 4.65 | 1.50 ± 0.35 (years) | 1.56 ± 0.37 (years) | BFK+rhIFN | rhIFN | 30 days | ④ |
| 2 | Wang (2019) | 40/40 | 47.9 ± 11. 4 | 47.8 ± 11. 2 | 12.4 ± 8.8 (months) | 12.3 ± 8.7 (months) | BFK+rhIFN | rhIFN | 3 months | ②③④ |
| 3 | Wang and Zhou (2021) | 60/60 | 35.26 ± 4.86 | 35.56 ± 5.10 | 7.51 ± 4.63 (months) | 7.69 ± 5.12 (months) | BFK+rhIFN | rhIFN | 30 days | ②③④ |
| 4 | Wang et al. (2021) | 29/29 | 35.28 ± 4.90 | 35.64 ± 4.83 | 5.26 ± 1.78 (months) | 5.13 ± 1.75 (months) | BFK+rhIFN | rhIFN | 6 weeks | ③ |
| 5 | Wu (2015) | 35/35 | 34.6 ± 4.1 | 33.9 ± 4.3 | 14.1 ± 3.3 (months) | 13.7 ± 3.1 (months) | BFK+rhIFN | rhIFN | 3 months | ③ |
| 6 | Wu and Zhang (2019) | 50/50 | 38.06 ± 5.39 | 37.55 ± 4.55 | 3.32 ± 1.25 (years) | 3.47 ± 1.13 (years) | BFK+rhIFN | rhIFN | 30 days | ②③④ |
| 7 | Xiao and Deng (2020) | 53/53 | 35.12 ± 6.11 | 34.98 ± 5.84 | 15.95 ± 4.65 (months) | 16.14 ± 5.22 (months) | BFK+rhIFN | rhIFN | 3 months | ②④ |
| 8 | Yang and Huang (2016) | 73/73 | 35.56 ± 4.39 | 35.79 ± 4.42 | 3.48 ± 1.25 (years) | 3.51 ± 1.19 (years) | BFK+rhIFN | rhIFN | 27 days | ②③ |
| 9 | Zhu (2018) | 33/33 | 47.37 ± 6.92 | 47.43 ± 6.84 | NR | NR | BFK+rhIFN | rhIFN | 30 days | ②③④ |
| 10 | Zhu and Xu (2021) | 60/60 | 22 to 55 | 23 to 54 | NR | NR | BFK+rhIFN | rhIFN | 27 days | ② |
| 11 | Chen (2021) | 49/48 | 34.33 ± 2.49 | 34.28 ± 2.54 | 15.59 ± 1.52 (months) | 15.48 ± 1.62 (months) | BFK+rhIFN | rhIFN | 30 days | ②③ |
| 12 | Chen and Huang (2022) | 120/120 | 40.22 ± 5.38 | 40.12 ± 5.46 | 7.39 ± 2.51 (months) | 7.55 ± 2.36 (months) | BFK+rhIFN | rhIFN | 30 days | ③④ |
| 13 | Cheng (2020) | 45/45 | 45.5 ± 9.5 | 41.2 ± 9.9 | 11.2 ± 3.3 (months) | 11.4 ± 3.7 (months) | BFK+rhIFN | rhIFN | 6 weeks | ③ |
| 14 | Gao (2018) | 38/34 | 35.45 ± 6.54 | 35.45 ± 6.54 | NR | NR | BFK+rhIFN | rhIFN | 6 weeks | ③ |
| 15 | Geng (2020) | 39/39 | 34.01 ± 4.72 | 33.85 ± 4.56 | NR | NR | BFK+rhIFN | rhIFN | 30 days | ②③④ |
| 16 | Han et al. (2019) | 50/50 | 38.71 ± 8.25 | 38.80 ± 8.14 | NR | NR | BFK+rhIFN | rhIFN | 3 months | ②③④ |
| 17 | He (2019) | 48/48 | 35.16 ± 6.29 | 34.85 ± 6.17 | 12.18 ± 3.52 (months) | 12.24 ± 3.49 (months) | BFK+rhIFN | rhIFN | 40 days | ②③④ |
| 18 | Lai et al. (2021) | 39/39 | 46.2 ± 3.5 | 46.2 ± 3.5 | NR | NR | BFK+rhIFN | rhIFN | 27 days | ①④ |
| 19 | Lai et al. (2022) | 40/40 | 35.12 ± 4.08 | 35.32 ± 4.31 | NR | NR | BFK+rhIFN | rhIFN | 48 days | ①④ |
| 20 | Li (2018) | 100/100 | 35.62 ± 2.19 | 35.92 ± 2.61 | NR | NR | BFK+rhIFN | rhIFN | 48 days | ③④ |

| No. | Study ID | Sample size/ Experiment/Control | Age(years, $\bar{x}$±s) | | Duration of disease($\bar{x}$±s) | | Intervention | | Intervention duration | Outcomes |
|---|---|---|---|---|---|---|---|---|---|---|
| | | | Experiment | Control | Experiment | Control | Experiment | Control | | |
| 21 | Li (2020) | 35/35 | 35.3 ± 5.0 | 36.3 ± 4.4 | 3.34 ± 0.63 (years) | 3.12 ± 0.57 (years) | BFK+rhIFN | rhIFN | 27 days | ②③④ |
| 22 | Li (2021) | 30/30 | 38.1 ± 5.4 | 38.1 ± 5.3 | 3.32 ± 1.24 (years) | 3.31 ± 1.23 (years) | BFK+rhIFN | rhIFN | 30 days | ①④ |
| 23 | Li et al. (2014) | 50/50 | NR | NR | NR | NR | BFK+rhIFN | rhIFN | 30 days | ④ |
| 24 | Li et al. (2018) | 73/73 | 35.56 ± 4.39 | 35.79 ± 4.42 | 3.48 ± 1.25 (years) | 3.51 ± 1.19 (years) | BFK+rhIFN | rhIFN | 27 days | ②③ |
| 25 | Liang et al. (2019) | 30/30 | 35.4 ± 4.2 | 35.6 ± 4.1 | 3.3 ± 1.1 (years) | 3.2 ± 1.1 (years) | BFK+rhIFN | rhIFN | 27 days | ① |
| 26 | Liu and Qi et al. (2016) | 112/109 | 36.13 ± 11.93 | 37.28 ± 12.36 | NR | NR | BFK+rhIFN | rhIFN | 3 months | ② |
| 27 | Liu and Huang et al. (2016) | 60/60 | 39.3 ± 4.1 | 39.23 ± 4.16 | NR | NR | BFK+rhIFN | rhIFN | 30 days | ②③④ |
| 28 | Ma et al. (2021) | 49/48 | 39.47 ± 6.01 | 39.47 ± 6.01 | NR | NR | BFK+rhIFN | rhIFN | 45 days | ④ |
| 29 | Mai et al. (2018) | 44/44 | 44.58 ± 4.25 | 45.16 ± 4.41 | 4.87 ± 1.62 (years) | 4.73 ± 1.84 (years) | BFK+rhIFN | rhIFN | 30 days | ② |
| 30 | Song et al. (2011) | 53/35 | 36.5 ± 0.5 | 37.4 ± 0.6 | NR | NR | BFK+rhIFN | rhIFN | 24 days | ④ |
| 31 | Su et al. (2020) | 91/82 | 49.91 ± 5.03 | 49.72 ± 4.84 | 18.30 ± 4.12 (months) | 18.24 ± 4.06 (months) | BFK+rhIFN | rhIFN | 3 months | ②④ |
| 32 | Li (2021) | 40/40 | 34.78±6.33 | 34.56±6.27 | NR | NR | BFK+rhIFN | rhIFN | 30 days | ② |
| 33 | Zhang (2016) | 49/49 | 34.78±6.33 | 34.56±6.27 | NR | NR | BFK+rhIFN | rhIFN | 21 days | ③ |
| 34 | Cao (2017) | 42/42 | 26.4 ± 3.0 | 26.2 ± 3.3 | 1.3 ± 0.5 (years) | 1.1 ± 0.2 (years) | SJZ+rhIFN | rhIFN | 54 days | ②③ |
| 35 | Han (2020) | 49/49 | 55.85 ± 8.99 | 56.45 ± 9.56 | 2.13 ± 0.12 (years) | 2.13 ± 0.13 (years) | SJZ+rhIFN | rhIFN | 54 days | ①③ |
| 36 | Hu (2018) | 90/90 | 35.67 ± 5.92 | 35.77 ± 5.93 | 2.18 ± 0.51 (years) | 2.16 ± 0.52 (years) | SJZ+rhIFN | rhIFN | 3 months | ②③ |
| 37 | Shen (2015) | 46/46 | 39.74 ± 4.74 | 40.32 ± 4.56 | 7.04 ± 1.47 (months) | 6.97 ± 1.52 (months) | SJZ+rhIFN | rhIFN | 3 months | ①③ |
| 38 | Wu and Feng (2018) | 53/53 | 40.26 ± 4.58 | 41.35 ± 4.54 | NR | NR | SJZ+rhIFN | rhIFN | 60 days | ③ |
| 39 | Pan and Zhang (2021) | 47/47 | 38.79 ± 4.22 | 38.86 ± 4.15 | 3.42 ± 1.09 (years) | 3.26 ± 1.06 (years) | KS+rhIFN | rhIFN | 27 days | ①③ |
| 40 | Zhao et al. (2016) | 40/40 | 18 to 60 | 18 to 60 | NR | NR | KS+rhIFN/KS | rhIFN | 3 months | ② |

| No. | Study ID | Sample size/ Experiment/Control | Age(years, $\overline{x}$±s) | | Duration of disease($\overline{x}$±s) | | Intervention | | Intervention duration | Outcomes |
|---|---|---|---|---|---|---|---|---|---|---|
| | | | Experiment | Control | Experiment | Control | Experiment | Control | | |
| 41 | Cheng (2021) | 60/60 | 34.4 ± 5.1 | 35.3 ± 4.3 | 7.1 ± 2.3 (months) | 7.5 ± 2.4 (months) | ZMK+rhIFN | rhIFN | 3 months | ②③ |
| 42 | Sun (2021) | 52/52 | 37.19 ± 9.51 | 37.28±9.56 | 13.71±2.71 (years) | 13.58±2.86 (years) | BFK+rhIFN | BFK | 2 months | ③④ |
| 43 | Ying (2019) | 62/62 | 37.2±4.6 | 38.4±4.7 | 1.8±0.8 (years) | 1.9±1.4 (years) | BFK+rhIFN | BFK | 54 days | ② |
| 44 | Du (2020) | 43/43 | 37.56±3.12 | 38.10±3.24 | 1.52±0.43 (years) | 1.63±0.39 (years) | BFK+rhIFN | BFK | 54 days | ②④ |
| 45 | Xu and Nie (2022) | 35/35 | 37.56±3.12 | 38.10±3.24 | 1.52±0.43 (years) | 1.63±0.39 (years) | BFK+rhIFN | BFK | 4 weeks | ②③④ |
| 46 | Ma (2019) | 41/41 | 23～61 | 25～59 | NR | NR | BFK+rhIFN | BFK | 21 days | ②③ |
| 47 | Hu (2017) | 58/58 | 42.19±3.82 | 43.76±2.06 | 16.57±4.32 (months) | 18.52±4.37 (months) | BFK+rhIFN | BFK | 64 days | ②④ |
| 48 | Xu (2016) | 66/54 | 37.03 ± 9.33 | 37. 03 ± 9. 33 | persistent high-risk HPV infection | persistent high-risk HPV infection | BFK | rhIFN | 3 months | ② |
| 49 | Zhang (2017) | 40/40 | 25～65 | 25～65 | NR | NR | BFK | rhIFN | 3 months | ① |
| 50 | Wu et al. (2018) | 55/55 | 20～53 | 20～53 | NR | NR | BFK | rhIFN | 6 weeks | ④ |
| 51 | Zhang (2019) | 51/51 | 38.57 ± 2.35 | 38.16±1.45 | NR | NR | BFK | rhIFN | 3 months | ②③④ |
| 52 | Ye et al. (2015) | 40/40 | 35.1 ± 5.1 | 34.8±4.7 | 3.6±1.0 (months) | 3.7±1.2 (months) | BFK | rhIFN | 6 weeks | ②④ |
| 53 | Huang and Chen (2012) | 20/20 | 26.3 ± 3.7 | 28.1±3.2 | NR | NR | SJZ | rhIFN | 60 days | ① |
| 54 | Huang et al. (2013) | 30/30 | 23～54 | 24～54.5 | NR | NR | SJZ | rhIFN | 60 days | ①③ |
| 55 | Li (2017) | 60/60 | 36.54±6.48 | 36.69±6.17 | NR | NR | SJZ | rhIFN | 60 days | ③ |
| 56 | Liu (2017) | 23/23 | 38.6±1.2 | 37.4±1.6 | NR | NR | SJZ | rhIFN | 60 days | ①③ |
| 57 | Qiu (2017) | 51/48 | 31.25±9.23 | s | NR | NR | SJZ | rhIFN | 60 days | ③ |
| 58 | Du (2018) | 30/30 | 29.42±4.31 | 29.39±4.28 | 5.42±1.27 (months) | 5.37±1.30 (months) | SJZ | rhIFN | 60 days | ②③ |
| 59 | Hua (2019) | 40/40 | 34.23±1.85 | 33.52±1.30 | NR | NR | SJZ | rhIFN | 20 days | ②③ |
| 60 | Zhang (2019)* | 40/40 | 35.6±2.6 | 36.9±3.5 | 8.6±2.0 (months) | 10.2±2.3 (months) | SJZ | rhIFN | 60 days | ③ |

NR = no detailed information; BFK = Baofukang suppository; SJZ = Compound seabuckthorn seed oil suppository; KS = Kushen gel; ZMK = Zhimikang suppository; rhIFN = recombinant human interferon. ①the

rate of HR-HPV clearance follow-up at 6 months; ②the rate of HR-HPV clearance after treatment; ③the clinical effectiveness rate; ④Adverse reactions.

**Supplementary S5: The product information (raw materials, labeled efficacy, indications, extraction procedure) of Chinese patent medicines**

| Name | Raw materials | Labeled efficacy | Indications | Extraction procedure |
|---|---|---|---|---|
| Baofukang suppository | Zedoary Turmeric Oil, Borneolum Syntheticum | Break the stasis of qi, build muscle and relieve pain. | It is used for the disease of subordination caused by dampness and heat stasis, the symptoms are large amount of banding, yellow color, and sometimes itching of the genitals; Mold vaginitis, senile vaginitis, cervical erosion see the above symptoms. | Flavor the oil and ice chips, add to the suitable ethanol, stir to dissolve. Take it separately Polytibial oxygen (40) stearate 1235g and polyethylene glycol 4000 200g, heated to melt. To transform, add macrogol 400 120g and laurazone 17.5g, stir well and add. The above liquid is stirred well, poured into the suppository mold, cooled and taken out to make 1000 capsules. |
| Compound seabuckthorn seed oil suppository | Seabuckthorn Seed Oil, Cnidii Fructus, Olibanum, Myrrha, Sophorae Fla Vescentis Radix, Calamina, Borneolum Syntheticum | Clear heat and dampness, reduce swelling and pain, kill insects and itch, and activate blood and muscle. | For cervical erosion caused by hot and humid bets. Symptoms: large amount of belt, yellow or yellow-white, bloody vaginal discharge or bleeding after sexual intercourse, vulvar itching, swelling and pain, waist and abdomen swelling, etc. | Crush the five flavors such as cnidii fructus, olibanum, myrrha, sophorae fla vescentis radix and calamina into the finest powder. The ice chips are finely ground, mixed with the above powder, and mixed. Take another glycerol gelatin matrix 1550g (gelatin 400g, glycerol 650g, boric acid 50g, distilled water 450g), heat and melt in a 75 °C water bath, add 150ml of sea buckthorn seed oil and water at the same temperature, quickly emulsify into a viscous gum, add the above powder, mix well, and pour it into the plug mold when the bubbles disappear, and after cooling, make 1000 capsules, which is obtained. |
| Kushen gel | Matrine | Clear heat and dampness, kill insects and relieve itching. | It is used for underband, pubic itching caused by humid heat bets. The symptoms are that the amount is large, thick as tofu dregs or yellow foam, its smell, vaginal flushing, swelling, vulvar pain and itching, even itching, frequent urination and astringency, bitter and sticky mouth, constipation or pond unpleasant, yellow urine; Mold vaginitis and trichomonas vaginitis are seen in the above symptoms. | Add 667ml of water to Matrine, add dilute hydrochloric acid dropwise, stir to dissolve, and adjust the pH value to 4 ~ 5 with dilute hydrochloric acid, and the solution is reserved; Take another 100g of glycerol and 30g of sodium carboxymethylcellulose, mix well, add Matrine while stirring, add an appropriate amount of water, mix well, and make 1000g, which is obtained. |

| | | | | |
|---|---|---|---|---|
| Zhimikang suppository | Phellodendri Chinensis Cortex, Sophorae Fla Vescentis Radix, Catechu, Aluminum Potassium Sulfate, Borneolum Syntheticum | Clear heat and detoxification, dry and wet convergence. | It is used for the disease caused by humid heat bets, the symptoms are large amount of banding, thick yellow color, odor, or dry stool; Bacterial vaginosis, trichomonas vaginosis, cervical erosion see the above symptoms. | Catechu and aluminum potassium sulfate crushed into fine powder; Borneolum syntheticum chips; Phellodendri chinensis cortex and sophorae fla vescentis radix participate in water decoction three times, the first time 2 hours, the second and third times 1 hour each, combined with decoction; Filtrate, the filtrate is concentrated to a clear paste with a relative density of 1.09 ~ 1.11 (80 ± 5 °C), ethanol is added to make the ethanol content 75%, stand to precipitate, take the supernatant to recover ethanol, oncentrate to an appropriate amount, spray dry, mix well with the above fine powder, sieve, add to the matrix made of olyoxyethylene monostearate cool 2000-2060g and glycerol 20ml, mix, perfusion, inject into the suppository mold, cool, make 1000 capsules. |

## Supplementary S6: Risk-of-bias judgments for the included RCTs

| Study ID | Random sequence generation | Allocation concealment | Blinding of participants and personnel | Blinding of outcome assessment | Incomplete outcome | Selective outcome reporting | Overall bias |
|---|---|---|---|---|---|---|---|
| Cao (2017) | Unclear | Unclear | High risk | Low risk | Low risk | Unclear | Low risk |
| Chen (2021) | Low risk | Unclear | High risk | Low risk | Low risk | Unclear | Low risk |
| Chen and Huang (2022) | Low risk | Unclear | High risk | Low risk | Low risk | Unclear | Low risk |
| Cheng (2020) | Low risk | Unclear | High risk | Low risk | Low risk | Unclear | Low risk |
| Cheng (2021) | Unclear | Unclear | High risk | Low risk | Low risk | Unclear | Low risk |
| Du (2018) | Unclear | Unclear | High risk | Low risk | Low risk | Unclear | Low risk |
| Du (2020) | Low risk | Unclear | High risk | Low risk | Low risk | Unclear | Low risk |
| Gao (2018) | Unclear | Unclear | High risk | Low risk | Low risk | Unclear | Low risk |
| Geng (2020) | Unclear | Unclear | High risk | Low risk | Low risk | Unclear | Low risk |
| Han (2020) | Low risk | Unclear | High risk | Low risk | Low risk | Unclear | Low risk |
| Han et al. (2019) | Low risk | Unclear | High risk | Low risk | Low risk | Unclear | Low risk |
| He (2019) | Low risk | Unclear | High risk | Low risk | Low risk | Unclear | Low risk |
| Hu (2017) | Unclear | Unclear | High risk | Low risk | Low risk | Unclear | Low risk |
| Hu (2018) | Low risk | Unclear | High risk | Low risk | Low risk | Unclear | Low risk |
| Hua (2019) | Unclear | Unclear | High risk | Low risk | Low risk | Unclear | Low risk |
| Huang and Chen (2012) | Unclear | Unclear | High risk | Low risk | Low risk | Unclear | Low risk |
| Huang et al. (2013) | Unclear | Unclear | High risk | Low risk | Low risk | Unclear | Low risk |
| Lai et al. (2019) | Unclear | Unclear | High risk | Low risk | Low risk | Unclear | Low risk |
| Lai et al. (2022) | Unclear | Unclear | High risk | Low risk | Low risk | Unclear | Low risk |
| Li (2017) | Unclear | Unclear | High risk | Low risk | Low risk | Unclear | Low risk |
| Li (2018) | Unclear | Unclear | High risk | Low risk | Low risk | Unclear | Low risk |
| Li (2020) | Low risk | Unclear | High risk | Low risk | Low risk | Unclear | Low risk |
| Li (2021) | Low risk | Unclear | High risk | Low risk | Low risk | Unclear | Low risk |
| Li (2021)* | Low risk | Unclear | High risk | Low risk | Low risk | Unclear | Low risk |
| Li et al. (2014) | Unclear | Unclear | High risk | Low risk | Low risk | Unclear | Low risk |
| Li et al. (2018) | Unclear | Unclear | High risk | Low risk | Low risk | Unclear | Low risk |
| Liang et al. (2019) | Unclear | Unclear | High risk | Low risk | Low risk | Unclear | Low risk |
| Liu (2017) | Unclear | Unclear | High risk | Low risk | Low risk | Unclear | Low risk |
| Liu and Huang et al. (2016) | Unclear | Unclear | High risk | Low risk | Low risk | Unclear | Low risk |
| Liu and Qi et al. (2016) | Unclear | Unclear | High risk | Low risk | Low risk | Unclear | Low risk |
| Ma (2019) | Low risk | Unclear | High risk | Low risk | Low risk | Unclear | Low risk |
| Ma et al. (2021) | Low risk | Unclear | High risk | Low risk | Low risk | Unclear | Low risk |
| Mai et al. (2018) | Low risk | Unclear | High risk | Low risk | Low risk | Unclear | Low risk |
| Pan and Zhang (2021) | Low risk | Unclear | High risk | Low risk | Low risk | Unclear | Low risk |
| Qiu (2017) | Low risk | Unclear | High risk | Low risk | Low risk | Unclear | Low risk |
| Shen (2015) | Unclear | Unclear | High risk | Low risk | Low risk | Unclear | Low risk |
| Song et al. (2011) | Unclear | Unclear | High risk | Low risk | Low risk | Unclear | Low risk |
| Su et al. (2020) | Low risk | Unclear | High risk | Low risk | Low risk | Unclear | Low risk |
| Sun (2021) | Low risk | Unclear | High risk | Low risk | Low risk | Unclear | Low risk |
| Tang and Tang et al. (2018) | Low risk | Unclear | High risk | Low risk | Low risk | Unclear | Low risk |
| Wang (2019) | Low risk | Unclear | High risk | Low risk | Low risk | Unclear | Low risk |
| Wang and Zhou (2021) | Low risk | Unclear | High risk | Low risk | Low risk | Unclear | Low risk |
| Wang et al. (2021) | Unclear | Unclear | High risk | Low risk | Low risk | Unclear | Low risk |
| Wu (2015) | Low risk | Unclear | High risk | Low risk | Low risk | Unclear | Low risk |
| Wu and Feng (2018) | Low risk | Unclear | High risk | Low risk | Low risk | Unclear | Low risk |
| Wu and Zhang (2019) | Low risk | Unclear | High risk | Low risk | Low risk | Unclear | Low risk |
| Wu et al. (2018) | Low risk | Unclear | High risk | Low risk | Low risk | Unclear | Low risk |
| Xiao and Deng (2020) | Low risk | Unclear | High risk | Low risk | Low risk | Unclear | Low risk |
| Xu (2016) | Unclear | Unclear | High risk | Low risk | Low risk | Unclear | Low risk |
| Xu and Nie (2022) | Low risk | Unclear | High risk | Low risk | Low risk | Unclear | Low risk |
| Yang and Huang (2016) | Low risk | Unclear | High risk | Low risk | Low risk | Unclear | Low risk |

| | | | | | | | |
|---|---|---|---|---|---|---|---|
| Ye et al. (2015) | Unclear | Unclear | High risk | Low risk | Low risk | Unclear | Low risk |
| Ying (2019) | Unclear | Unclear | High risk | Low risk | Low risk | Unclear | Low risk |
| Zhang (2016) | Low risk | Unclear | High risk | Low risk | Low risk | Unclear | Low risk |
| Zhang (2017) | Unclear | Unclear | High risk | Low risk | Low risk | Unclear | Low risk |
| Zhang (2019) | Unclear | Unclear | High risk | Low risk | Low risk | Unclear | Low risk |
| Zhang (2019)* | Unclear | Unclear | High risk | Low risk | Low risk | Unclear | Low risk |
| Zhao et al. (2016) | Unclear | Unclear | High risk | Low risk | Low risk | Unclear | Low risk |
| Zhu (2018) | Low risk | Unclear | High risk | Low risk | Low risk | Unclear | Low risk |
| Zhu and Xu (2021) | Low risk | Unclear | High risk | Low risk | Low risk | Unclear | Low risk |

**Supplementary S7: GRADE assessment for NMA—the rate of HR-HPV clearance follow-up at 6 months**

GRADE (grading of recommendations assessment, development, and evaluation) working group grades of evidence (or certainty of evidence): high quality—very confident true effect lies close to that of estimate of effect; moderate quality—moderately confident in effect estimate; true effect is likely to be close to estimate of effect, but there is a possibility that it is substantially different; low quality—confidence in effect estimate is limited; true effect may be substantially different from estimate of effect; very low quality: very little confidence in effect estimate; true effect is likely to be substantially different from estimate of effect.

| ntion | Comparator | Direct evidence | | Indirect evidence | | Network meta-analysis | |
|---|---|---|---|---|---|---|---|
| | | Odds Ratio (95% CrI) | Quality of evidence | Odds Ratio (95% CrI) | Quality of evidence | Odds Ratio (95% CrI) | Final network rating |
| BFK+rhIFN | rhIFN | 3.4 (0.9, 15.5) | Very low | — | — | 3.4 (0.9, 15.5) | Very low |
| SJZ+rhIFN | rhIFN | 3.2 (0.4, 25.8) | Very low | — | — | 3.2 (0.4, 25.8) | Very low |
| KS+rhIFN | rhIFN | 3.2 (0.2, 55.5) | Very low | — | — | 3.2 (0.2, 55.5) | Very low |
| BFK | rhIFN | 0.4 (0.02, 8.4) | Very low | — | — | 0.4 (0.02, 8.4) | Very low |
| SJZ | rhIFN | 0.1 (0.01, 0.6) | Very low | — | — | 0.1 (0.01, 0.6) | Very low |
| BFK+rhIFN | SJZ | — | — | 36.5 (4.0, 570.1) | Very low | 36.5 (4.0, 570.1) | Very low |
| SJZ+rhIFN | SJZ | — | — | 34.9 (2.5, 753.5) | Very low | 34.9 (2.5, 753.5) | Very low |
| KS+rhIFN | SJZ | — | — | 34.5 (1.3, 1319.2) | Very low | 34.5 (1.3, 1319.2) | Very low |
| BFK | SJZ | — | — | 4.5 (0.1, 195.4) | Very low | 4.5 (0.1, 195.4) | Very low |
| BFK+rhIFN | BFK | — | — | 8.1 (0.3, 248.3) | Very low | 8.1 (0.3, 248.3) | Very low |
| SJZ+rhIFN | BFK | — | — | 7.8 (0.4, 149.7) | Very low | 7.8 (0.4, 149.7) | Very low |
| KS+rhIFN | BFK | — | — | 7.7 (0.2, 302.0) | Very low | 7.7 (0.2, 302.0) | Very low |
| BFK+rhIFN | KS+rhIFN | — | — | 7.6 (0.1, 496.1) | Very low | 7.6 (0.1, 496.1) | Very low |
| SJZ+rhIFN | KS+rhIFN | — | — | 1.0 (0.03, 35.9) | Very low | 1.0 (0.03, 35.9) | Very low |
| BFK+rhIFN | SJZ+rhIFN | — | — | 1.0 (0.1, 13.9) | Very low | 1.0 (0.1, 13.9) | Very low |

CrI = credible intervals; BFK = Baofukang suppository; SJZ = Compound seabuckthorn seed oil suppository; KS = Kushen gel; ZMK = Zhimikang suppository; rhIFN

= recombinant human interferon.

**Supplementary S8: GRADE assessment for NMA—the rate of HR-HPV clearance after treatment**

GRADE (grading of recommendations assessment, development, and evaluation) working group grades of evidence (or certainty of evidence): high quality—very confident true effect lies close to that of estimate of effect; moderate quality—moderately confident in effect estimate; true effect is likely to be close to estimate of effect, but there is a possibility that it is substantially different; low quality—confidence in effect estimate is limited; true effect may be substantially different from estimate of effect; very low quality: very little confidence in effect estimate; true effect is likely to be substantially different from estimate of effect.

| Intervention | Comparator | Direct evidence | | Indirect evidence | | Network meta-analysis | |
|---|---|---|---|---|---|---|---|
| | | Odds Ratio (95% CrI) | Quality of evidence | Odds Ratio (95% CrI) | Quality of evidence | Odds Ratio (95% CrI) | Final network rating |
| BFK+rhIFN | rhIFN | 3.5 (2.7, 4.6) | Low | 2.8 (1.4, 5.7) | Low | 3.7 (2.8, 4.9) | Low |
| SJZ+rhIFN | rhIFN | 3.5 (1.4, 9.3) | Very low | — | — | 3.5 (1.4, 9.3) | Very low |
| KS+rhIFN | rhIFN | 6.9 (2.0, 26.2) | Very low | — | — | 6.9 (2.0, 26.2) | Very low |
| ZMK+rhIFN | rhIFN | 2.5 (0.8, 7.9) | Very low | — | — | 2.5 (0.8, 7.9) | Very low |
| BFK | rhIFN | 0.9 (0.5, 1.6) | Low | 1.2 (0.7, 2.0) | Low | 1.0 (0.7, 1.5) | Low |
| SJZ | rhIFN | 0.1 (0.03, 0.4) | Very low | — | — | 0.1 (0.03, 0.4) | Very low |
| KS | rhIFN | 3.0 (0.9, 10.9) | Very low | — | — | 3.0 (0.9, 10.9) | Very low |
| BFK+rhIFN | SJZ | — | — | 33.0 (9.4, 141.6) | Very low | 33.0 (9.4, 141.6) | Very low |
| SJZ+rhIFN | SJZ | — | — | 31.9 (6.6, 177.5) | Very low | 31.9 (6.6, 177.5) | Very low |
| KS+rhIFN | SJZ | — | — | 63.0 (10.8, 453.5) | Very low | 63.0 (10.8, 453.5) | Very low |
| ZMK+rhIFN | SJZ | — | — | 23.0 (4.3, 137.0) | Very low | 23.0 (4.3, 137.0) | Very low |
| BFK | SJZ | — | — | 9.1 (2.5, 39.3) | Very low | 9.1 (2.5, 39.3) | Very low |
| KS | SJZ | — | — | 27.4 (4.6, 184.7) | Very low | 27.4 (4.6, 184.7) | Very low |
| BFK+rhIFN | BFK | 3.2 (1.9, 5.3) | Low | 3.8 (2.4, 4.3) | Low | 3.6 (2.5, 5.4) | Low |

| | | | | | | | |
|---|---|---|---|---|---|---|---|
| SJZ+rhIFN | BFK | — | — | 3.5 (1.3, 10.0) | Very low | 3.5 (1.3, 10.0) | Very low |
| KS+rhIFN | BFK | — | — | 6.9 (1.9, 27.3) | Very low | 6.9 (1.9, 27.3) | Very low |
| ZMK+rhIFN | BFK | — | — | 2.5 (0.8, 8.4) | Very low | 2.5 (0.8, 8.4) | Very low |
| KS | BFK | — | — | 3.0 (0.8, 11.4) | Very low | 3.0 (0.8, 11.4) | Very low |
| BFK+rhIFN | ZMK+rhIFN | — | — | 1.4 (0.5, 4.6) | Very low | 1.4 (0.5, 4.6) | Very low |
| SJZ+rhIFN | ZMK+rhIFN | — | — | 1.4 (0.3, 6.1) | Very low | 1.4 (0.3, 6.1) | Very low |
| KS+rhIFN | ZMK+rhIFN | — | — | 2.7 (0.5, 15.2) | Very low | 2.7 (0.5, 15.2) | Very low |
| KS | ZMK+rhIFN | — | — | 1.2 (0.2, 6.4) | Very low | 1.2 (0.2, 6.4) | Very low |
| BFK+rhIFN | KS | — | — | 1.2 (0.3, 4.4) | Very low | 1.2 (0.3, 4.4) | Very low |
| SJZ+rhIFN | KS | — | — | 1.2 (0.2, 5.6) | Very low | 1.2 (0.2, 5.6) | Very low |
| KS+rhIFN | KS | — | — | 2.3 (0.4, 14.3) | Very low | 2.3 (0.4, 14.3) | Very low |
| BFK+rhIFN | SJZ+rhIFN | — | — | 1.0 (0.4, 2.8) | Very low | 1.0 (0.4, 2.8) | Very low |
| KS+rhIFN | SJZ+rhIFN | — | — | 2.0 (0.4, 10.0) | Very low | 2.0 (0.4, 10.0) | Very low |
| KS+rhIFN | BFK+rhIFN | — | — | 1.9 (0.5, 7.3) | Very low | 1.9 (0.5, 7.3) | Very low |

CrI = credible intervals; BFK = Baofukang suppository; SJZ = Compound seabuckthorn seed oil suppository; KS = Kushen gel; ZMK = Zhimikang suppository; rhIFN = recombinant human interferon.

**Supplementary S9: GRADE assessment for NMA—the clinical effectiveness rate**

GRADE (grading of recommendations assessment, development, and evaluation) working group grades of evidence (or certainty of evidence): high quality—very confident true effect lies close to that of estimate of effect; moderate quality—moderately confident in effect estimate; true effect is likely to be close to estimate of effect, but there is a possibility that it is substantially different; low quality— confidence in effect estimate is limited; true effect may be substantially different from estimate of effect; very low quality: very little confidence in effect estimate; true effect is likely to be substantially different from estimate of effect.

| Intervention | Comparator | Direct evidence | | Indirect evidence | | Network meta-analysis | |
|---|---|---|---|---|---|---|---|
| | | Odds Ratio (95% CrI) | Quality of evidence | Odds Ratio (95% CrI) | Quality of evidence | Odds Ratio (95% CrI) | Final network rating |
| BFK+rhIFN | rhIFN | 2.6 (2.1, 3.2) | Moderate | 2.1 (0.8, 5.6) | Low | 2.6 (2.1, 3.2) | Low |
| SJZ+rhIFN | rhIFN | 1.8 (1.2, 2.7) | Low | — | — | 1.8 (1.2, 2.7) | Low |
| KS+rhIFN | rhIFN | 3.7 (1.2, 12.2) | Very low | — | — | 3.7 (1.2, 12.2) | Very low |
| ZMK+rhIFN | rhIFN | 2.6 (1.0, 6.8) | Very low | — | — | 2.6 (1.0, 6.8) | Very low |
| BFK | rhIFN | 1.0 (0.6, 1.5) | Very low | 1.0 (0.6, 1.9) | Very low | 1.0 (0.6, 1.5) | Very low |
| SJZ | rhIFN | 0.2 (0.1, 0.4) | Low | — | — | 0.2 (0.1, 0.4) | Low |
| BFK+rhIFN | SJZ | — | — | 12.4 (6.6, 24.7) | Low | 12.4 (6.6, 24.7) | Very low |
| SJZ+rhIFN | SJZ | — | — | 8.6 (4.1, 18.4) | Low | 8.6 (4.1, 18.4) | Very low |
| KS+rhIFN | SJZ | — | — | 17.8 (4.9, 68.4) | Very low | 17.8 (4.9, 68.4) | Very low |
| ZMK+rhIFN | SJZ | — | — | 12.5 (4.2, 40.0) | Very low | 12.5 (4.2, 40.0) | Very low |
| BFK | SJZ | — | — | 4.8 (2.3, 10.1) | Very low | 4.8 (2.3, 10.1) | Very low |
| BFK+rhIFN | BFK | 2.5 (1.4, 4.4) | Low | 2.7 (1.6, 3.9) | Very low | 2.6 (1.7, 4.1) | Very low |
| SJZ+rhIFN | BFK | — | — | 1.8 (0.9, 3.4) | Very low | 1.8 (0.9, 3.4) | Very low |
| KS+rhIFN | BFK | — | — | 3.7 (1.1, 13.5) | Very low | 3.7 (1.1, 13.5) | Very low |
| ZMK+rhIFN | BFK | — | — | 2.6 (0.9, 7.6) | Very low | 2.6 (0.9, 7.6) | Very low |
| BFK+rhIFN | ZMK+rhIFN | — | — | 1.0 (0.4, 2.6) | Very low | 1.0 (0.4, 2.6) | Very low |

| | | | | | | | |
|---|---|---|---|---|---|---|---|
| SJZ+rhIFN | ZMK+rhIFN | — | — | 0.7 (0.2, 1.9) | Very low | 0.7 (0.2, 1.9) | Very low |
| KS+rhIFN | ZMK+rhIFN | — | — | 1.4 (0.3, 6.7) | Very low | 1.4 (0.3, 6.7) | Very low |
| BFK+rhIFN | SJZ+rhIFN | — | — | 1.5 (0.9, 2.4) | Low | 1.5 (0.9, 2.4) | Low |
| KS+rhIFN | SJZ+rhIFN | — | — | 2.1 (0.6, 7.4) | Very low | 2.1 (0.6, 7.4) | Very low |
| KS+rhIFN | BFK+rhIFN | — | — | 1.4 (0.5, 4.9) | Very low | 1.4 (0.5, 4.9) | Very low |

CrI = credible intervals; BFK = Baofukang suppository; SJZ = Compound seabuckthorn seed oil suppository; KS = Kushen gel; ZMK = Zhimikang suppository; rhIFN = recombinant human interferon.

**Supplementary S10: DIC values and I-square of fixed-effect model and random-effect model**

| **Outcomes** | **Model** | **DIC** | **I-square** |
|---|---|---|---|
| the rate of HR-HPV clearance follow-up at 6 months | fixed- effect | 72.51 | 48% |
| | random-effect | 53.29 | 3% |
| the rate of HR-HPV clearance after treatment | fixed- effect | 123.15 | 14% |
| | random-effect | 119.86 | 0% |
| the clinical effectiveness rate | fixed- effect | 145.27 | 14% |
| | random-effect | 145.63 | 7% |

**Supplementary S11: Ranking probabilities and SUCRA values**

| | BFK+rhIFN | SJZ+rhIFN | KS+rhIFN | ZMK+rhIFN | BFK | SJZ | KS | rhIFN |
|---|---|---|---|---|---|---|---|---|
| **The rate of HR-HPV clearance follow-up at 6 months** | | | | | | | | |
| Best | 0.29 | 0.31 | 0.36 | — | 0.03 | 0.00 | — | 0.00 |
| 2nd | 0.39 | 0.31 | 0.23 | — | 0.05 | 0.00 | — | 0.02 |
| 3rd | 0.26 | 0.26 | 0.22 | — | 0.08 | 0.00 | — | 0.18 |
| 4th | 0.04 | 0.08 | 0.11 | — | 0.14 | 0.01 | — | 0.62 |
| 5th | 0.02 | 0.04 | 0.07 | — | 0.53 | 0.17 | — | 0.18 |
| Worst | 0.00 | 0.00 | 0.01 | — | 0.17 | 0.82 | — | 0.00 |
| SUCRA (%) | 78.16 | 75.4 | 73.17 | — | 28.16 | 4.20 | — | 40.9 |
| **The rate of HR-HPV clearance after treatment** | | | | | | | | |
| Best | 0.05 | 0.12 | 0.66 | 0.05 | 0.00 | 0.00 | 0.12 | 0.00 |
| 2nd | 0.28 | 0.25 | 0.16 | 0.12 | 0.00 | 0.00 | 0.18 | 0.00 |
| 3rd | 0.39 | 0.23 | 0.08 | 0.14 | 0.00 | 0.00 | 0.16 | 0.00 |
| 4th | 0.23 | 0.24 | 0.06 | 0.24 | 0.01 | 0.00 | 0.22 | 0.00 |
| 5th | 0.05 | 0.16 | 0.04 | 0.38 | 0.08 | 0.00 | 0.27 | 0.05 |
| 6th | 0.00 | 0.00 | 0.00 | 0.03 | 0.46 | 0.00 | 0.02 | 0.48 |
| 7th | 0.00 | 0.00 | 0.00 | 0.04 | 0.45 | 0.01 | 0.03 | 0.47 |
| Worst | 0.00 | 0.00 | 0.00 | 0.00 | 0.00 | 0.99 | 0.00 | 0.00 |
| SUCRA (%) | 72.15 | 70.04 | 90.77 | 57.26 | 23.39 | 0.01 | 63.86 | 22.5 |

| | | | | | | | | |
|---|---|---|---|---|---|---|---|---|
| **The clinical effectiveness rate** | | | | | | | | |
| Best | 0.14 | 0.01 | 0.60 | 0.25 | 0.00 | 0.00 | — | 0.00 |
| 2nd | 0.46 | 0.06 | 0.17 | 0.30 | 0.00 | 0.00 | — | 0.00 |
| 3rd | 0.37 | 0.26 | 0.13 | 0.24 | 0.01 | 0.00 | — | 0.00 |
| 4th | 0.03 | 0.64 | 0.08 | 0.17 | 0.06 | 0.00 | — | 0.02 |
| 5th | 0.00 | 0.03 | 0.01 | 0.02 | 0.43 | 0.00 | — | 0.51 |
| 6th | 0.00 | 0.00 | 0.01 | 0.02 | 0.50 | 0.01 | — | 0.47 |
| Worst | 0.00 | 0.00 | 0.00 | 0.00 | 0.00 | 0.99 | — | 0.00 |
| SUCRA (%) | 78.61 | 56.19 | 87.39 | 75.78 | 26.12 | 0.00 | — | 25.91 |

Ranking probabilities were estimated using a parametric bootstrap procedure with 10,000 resamples. SUCRA = Surface Under the Cumulative Ranking curve; BFK = Baofukang suppository; SJZ = Compound seabuckthorn seed oil suppository; KS = Kushen gel; ZMK = Zhimikang suppository; rhIFN = recombinant human interferon.

**Supplementary S12: The detailed adverse reactions of the included studies**

| No. | Study ID | Experiment | | | Control | | |
|---|---|---|---|---|---|---|---|
| | | Intervention | Adverse reactions | events/ totals (proportion %) | Intervention | Adverse reactions | events/ totals (proportion %) |
| **1** | Tang and Tang et al. (2018) | BFK+rhIFN | vagina hot; secretion increase; leukorrhea abnormal | 4/20 (20%) | rhIFN | secretion increase; leukorrhea abnormal | 3/20 (15%) |
| **2** | Wang (2019) | BFK+rhIFN | Vaginal dryness; secretion increase | 2/40 (5%) | rhIFN | muscle aches; secretion increase | 3/40 (7.5%) |
| **3** | Wang and Zhou (2021) | BFK+rhIFN | secretion increase; leukorrhea abnormal; vaginal discomfort | 10/60 (8.33%) | rhIFN | secretion increase; leukorrhea abnormal; vaginal discomfort | 13/60 (21.67%) |
| **4** | Wu and Zhang (2019) | BFK+rhIFN | vagina hot | 2/50 (4%) | rhIFN | vagina hot | 3/50 (6%) |
| **5** | Xiao and Deng (2020) | BFK+rhIFN | vagina hot; Itching of the vulva | 3/53 (5.66%) | rhIFN | vagina hot; Itching of the vulva | 5/53 (9.43%) |
| **6** | Zhu (2018) | BFK+rhIFN | vagina hot; muscle aches | 2/33 (6.06%) | rhIFN | vaginal dryness | 1/33 (3.03%) |
| **7** | Geng (2020) | BFK+rhIFN | gastrointestinal discomfort; skin allergic; Itching of the vulva | 5/39 (12.82%) | rhIFN | gastrointestinal discomfort; skin allergic; Itching of the vulva | 3/39 (7.69%) |
| **8** | Han et al. (2019) | BFK+rhIFN | vagina hot | 3/50 (6%) | rhIFN | vagina hot | 2/50 (4%) |
| **9** | He (2019) | BFK+rhIFN | vaginal dryness; vaginal redness and swelling; vaginal itching | 4/48 (8.33%) | rhIFN | vaginal dryness; vaginal itching | 6/48 (12.50%) |
| **10** | Lai et al. (2021) | BFK+rhIFN | vagina hot; vaginal dryness | 5/39 (12.82%) | rhIFN | vagina hot; vaginal dryness | 4/39 (10.26%) |
| **11** | Lai et al. (2022) | BFK+rhIFN | vaginal discomfort; vagina hot | 2/40 (5%) | rhIFN | vaginal discomfort; vagina hot; frequent urination | 6/40 (15%) |
| **12** | Li (2018) | BFK+rhIFN | vaginal discomfort; vagina hot; frequent urination | 9/100 (9%) | rhIFN | vaginal discomfort; vagina hot; frequent urination | 11/100 (11%) |
| **13** | Li (2020) | BFK+rhIFN | secretion increase; rash | 2/ 35 (5.71%) | rhIFN | secretion increase; rash | 3/35 (8.57%) |
| **14** | Li (2021) | BFK+rhIFN | chills | 1/30 (3.3%) | rhIFN | secretion increase | 2/30 (6.7%) |
| **15** | Li et al. (2014) | BFK+rhIFN | itching of the vulva | 2/50 (4%) | rhIFN | nothing | 0/50 (0) |
| **16** | Liu and Huang et al. (2016) | BFK+rhIFN | skin allergies | 1/60 (1.67%) | rhIFN | gastrointestinal discomfort; skin allergies | 2/60 (3.33%) |
| **17** | Ma et al. (2021) | BFK+rhIFN | fever | 1/49 (2.04%) | rhIFN | lower abdomen swelling | 1/48 (2.08%) |
| **18** | Su et al. (2020) | BFK+rhIFN | vagina hot; secretion increase; leukorrhea abnormal | 10/91 (10.99%) | rhIFN | secretion increase; leukorrhea abnormal; vaginal itching | 13/82 (15.85%) |

| No. | Study ID | Experiment | | | Control | | |
|---|---|---|---|---|---|---|---|
| | | Intervention | Adverse reactions | events/ totals (proportion %) | Intervention | Adverse reactions | events/ totals (proportion %) |
| **19** | Song et al. (2011) | BFK+rhIFN | nothing | 0/53 (0) | rhIFN | nothing | 0/35 (0) |
| **20** | Chen and Huang (2022) | BFK+rhIFN | fever; vaginal dryness; vaginal itching | 11/120 (9.16%) | rhIFN | fever; vaginal dryness; vaginal itching | 8/120 (6.67%) |
| **21** | Sun (2021) | BFK+rhIFN | no detailed information | 5/52 (9.62%) | BFK | no detailed information | 8/52 (15.38%) |
| **22** | Du (2020) | BFK+rhIFN | vagina hot; vaginal itching | 2/43 (4.65%) | BFK | vagina hot; vaginal itching | 4/43 (9.3%) |
| **23** | Xu and Nie (2022) | BFK+rhIFN | nausea and vomiting | 2/35 (5.71%) | BFK | nausea and vomiting | 2/35 (5.71%) |
| **24** | Hu (2017) | BFK+rhIFN | fever; secretion increase; rash; soreness in the lower back | 9/58 (15.52%) | BFK | fever; secretion increase; rash; | 10/58 (17.24%) |
| **25** | Wu et al. (2018) | BFK | vaginal itching; vaginal edema | 1/55 (1.8%) | rhIFN | vaginal itching; vaginal edema | 2/55 (3.6%) |
| **26** | Zhang (2019) | BFK | nothing | 0/51 (0) | rhIFN | nothing | 0/51 (0) |
| **27** | Ye et al. (2015) | BFK | nothing | 0/40 (0) | rhIFN | nothing | 0/40 (0) |

BFK = Baofukang suppository; rhIFN = recombinant human interferon.

1.The rate of hr-HPV clearance after treatment

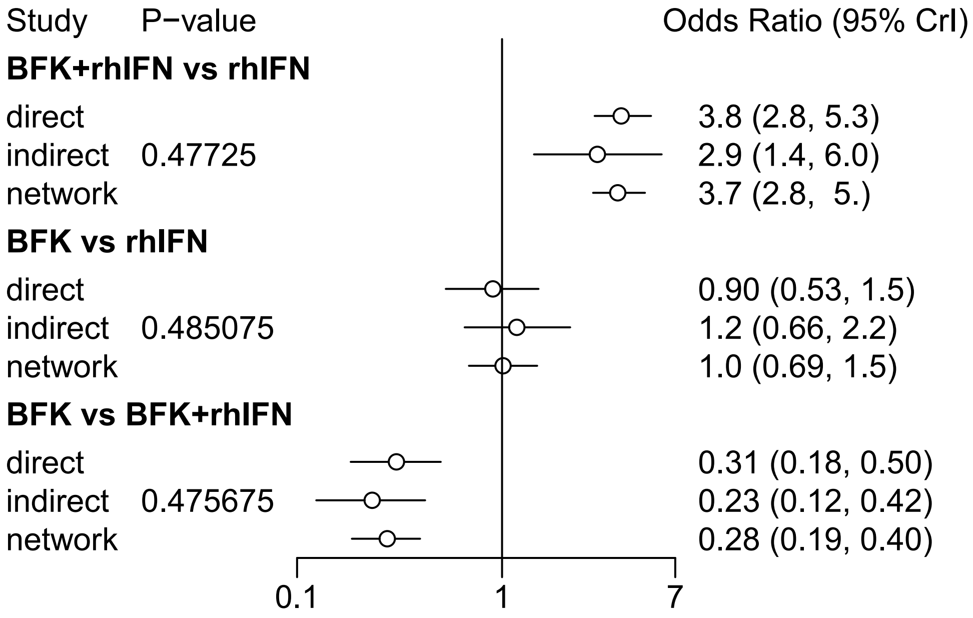


2.The clinical effectiveness rate

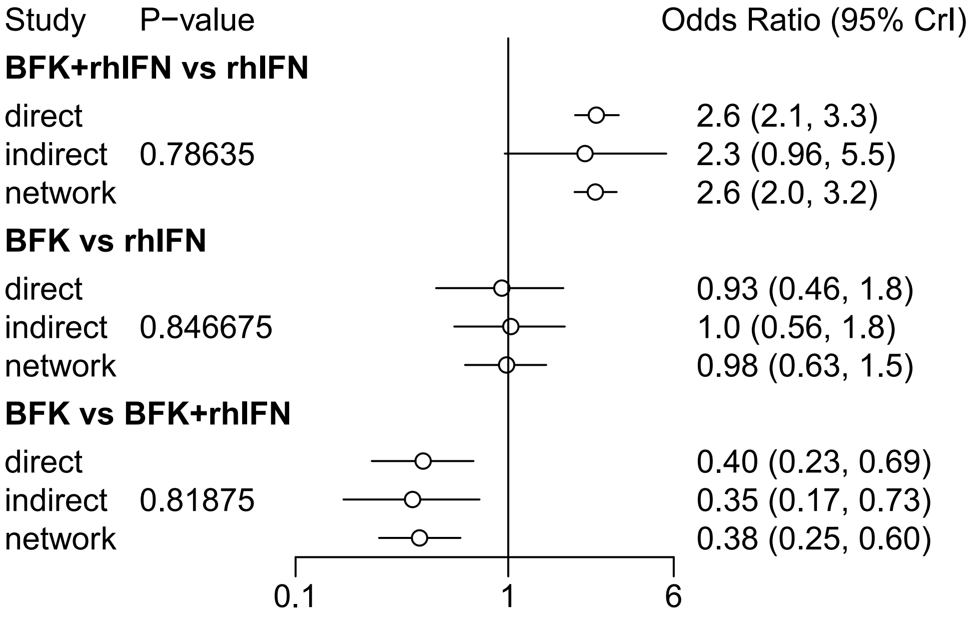

Supplement: Supplementary file 1 — Supplementary information and tables. [file jcav14p2373s1.pdf]
